# Exploring the Effects of Qigong, Tai Chi, and Yoga on Fatigue, Mental Health, and Sleep Quality in Chronic Fatigue and Post-COVID Syndromes: A Systematic Review with Meta-Analysis

**DOI:** 10.3390/healthcare12202020

**Published:** 2024-10-11

**Authors:** Hermann Fricke-Comellas, Alberto Marcos Heredia-Rizo, María Jesús Casuso-Holgado, Jesús Salas-González, Lourdes María Fernández-Seguín

**Affiliations:** 1 Departamento de Fisioterapia, Facultad de Enfermería, Fisioterapia y Podología, Universidad de Sevilla, 41009 Sevilla, Spain; hfricke@us.es (H.F.-C.); jessalgon1@alum.us.es (J.S.-G.); 2 CTS 1110: Understanding Movement and Self in Health from Science (UMSS) Research Group, 41009 Andalusia, Spain; mcasuso@us.es (M.J.C.-H.); lfdez@us.es (L.M.F.-S.); 3 Instituto de Biomedicina de Sevilla, IBiS, Departamento de Fisioterapia, Universidad de Sevilla, 41013 Seville, Spain

**Keywords:** exercise movement techniques, fatigue syndrome, chronic, post-acute COVID-19 syndrome, mind–body therapies, qigong, tai ji, yoga

## Abstract

**Background/Objectives**: Chronic fatigue syndrome (CFS) and post-COVID syndrome (PCS) pose a substantial socioeconomic burden. The aim of this systematic review was to assess current evidence regarding the effect of the most popular forms of movement-based mindful exercises, i.e., qigong, tai chi, and yoga, on fatigue and associated symptoms in CFS and PCS. **Methods**: CINAHL, Embase, PsycINFO, PubMed, Scopus, and the Cochrane Library were searched from inception to October 2023. Randomized controlled trials (RCTs) where qigong, tai chi, or yoga were compared with waitlist, no intervention, or active controls were included. Independent reviewers participated in data extraction, and evaluated risk of bias, spin of information, completeness of intervention description, and certainty of the evidence (GRADE). Meta-analyses were conducted. The primary outcome was the level of fatigue. Secondary measures were the severity of anxiety and depressive symptoms and sleep quality. Results were expressed as mean difference (MD) or standardized mean difference (SMD) with a 95% confidence interval (CI). **Results**: Thirteen RCTs with 661 participants were included, with most studies presenting a moderate or high risk of bias. Mindful exercises were more effective than control interventions to alleviate fatigue: SMD (95%CI) = −0.44 (−0.63 to −0.25), I^2^ = 48%, *p* < 0.0001. Positive effects were also observed for secondary outcomes. The certainty of the evidence was low or very low. **Conclusions**: Qigong, tai chi, and yoga may be effective to reduce fatigue and improve anxiety, depression, and sleep quality in adults with CFS or PCS. However, serious methodological concerns limit the clinical applicability of these findings.

## 1. Introduction

Experiencing fatigue, whether physical or mental, is common in up to 40% of adults [1]. When unexplained fatigue persists for more than 6 months and is accompanied by disability and a range of limiting symptoms, including headaches, poor sleep, cognitive impairments, and pain, it is categorized as chronic fatigue syndrome / myalgic encephalitis (CFS) [2,3,4]. In developed countries, the prevalence of CFS ranges between 0.2 and 1%, with approximately 2 million diagnosed cases in Europe [5] and 2.5 million in the US, although a considerable number of individuals still remain undiagnosed [6]. Therefore, the socioeconomic burden of CFS has been insufficiently documented [7], despite evidence suggesting substantial productivity losses [5,7]. Medical conditions characterized by fatigue, post-exertional malaise, and pain have been studied over the years, and have gained special attention since the COVID-19 pandemic and the onset of the post-COVID syndrome (PCS) [8]. As in CFS, autoimmune-mediated dysfunctions of the nervous system seem to contribute to PCS [8]. The World Health Organization classifies CFS as a post-viral fatigue syndrome [9], whereas PCS is considered a condition of uncertain etiology [10]. The prevalence of the most common PCS-related symptoms, such as fatigue, dyspnea, and cognitive issues, is estimated to be 50% in community settings [11]. Both CFS and PCS are systemic illnesses triggered by infection, sharing similar symptoms and pathological mechanisms [12,13,14]. This comparable clinical phenotype includes the psychological sphere, i.e., an increased severity of anxiety and depression, especially in those with a combined diagnosis of CFS and PCS [15]. This raises the question of whether these two conditions may represent manifestations of a more encompassing illness [13].

Physical activity and exercise, core competencies of professionals in physical medicine and physiotherapy when applied to the treatment of medical conditions, are often recommended to manage symptoms in individuals with CFS [16] or PCS [17]. Aerobic exercises, such as walking, or cycling, have shown positive results on fatigue, physical function, and sleep quality compared to usual care in those with CFS, although the effects on pain, quality of life, and mental health remain uncertain [18]. The impact of exercise-based rehabilitation in adults with PCS is not well understood yet, with concerns raised about the potential exacerbation of post-exertional symptoms by graded exercise [17]. Individually tailored exercise programs are considered safe and essential to integrate patients with PCS back into society [17,19]. Among various forms of exercise practice, movement-based mindful exercises represent unique types of low-impact, low-intensity physical activity. Also known as mind–body exercises, this form of multimodal activity is characterized by slow, intentional movements, full-body stretching, controlled breathing, relaxation, and focused mental attention or a meditative state [20,21,22]. Mind–body exercises have shown benefits for both physical and mental health [23,24]. The most common forms were selected, i.e., qigong, tai chi, and yoga [23,24], as they are often grouped together in the scientific literature [22]. Various systematic reviews have explored the efficacy of qigong or tai chi for chronic fatigue [25,26,27], but none included yoga interventions or patients with PCS. The aim of this systematic review with meta-analysis was to evaluate randomized controlled trials (RCTs) where qigong, tai chi, or yoga were used to manage fatigue and associated symptoms in individuals diagnosed with CFS or PCS.

## 2. Materials and Methods

The study followed the principles of the Preferred Reporting Items for Systematic Reviews and Meta-Analyses (PRISMA) 2020 statement and the PRISMA for abstracts. The review protocol was prospectively registered at Open Science Framework (doi: 10.17605/OSF.IO/5VF9U). There were no deviations from the intended protocol.

### 2.1. Data Sources and Search Strategies

One researcher (HFC) searched CINAHL (via EBSCOhost), Embase, PsycINFO (via EBSCOhost), PubMed, Scopus, and the Cochrane Library from inception to October 2023. Search filters for document type were applied where possible. The search syntax was as follows: (“post infection syndromes” OR “chronic fatigue syndrome” OR “myalgic encephalomyelitis” OR “long covid” OR “post covid condition” OR “postviral fatigue syndrome”) AND (“qigong” OR “qi gong” OR “tai” OR “t’ai” OR “taiji” OR “yoga” OR “pranayama” OR “chi kung” OR “baduanjin” OR “wuqinxi” OR “liuzijue” OR “yijinjing” OR “exercise” OR “training” OR “Pilates” OR “breathing exercises”) AND “trial”. Manual searches were conducted in the list of references of reviews found in the electronic databases. The complete search strategies are detailed in Supplementary File S1.

### 2.2. Eligibility Criteria

The eligibility criteria were defined according to the PICOs framework. We included RCTs that assessed the effects of qigong, tai chi, yoga, and their variants, in adults diagnosed with CFS or PCS. Control groups were waitlist, no intervention, or active controls. The diagnosis of CFS should adhere to established guidelines, as proposed by Fukuda et al. (1994) [2], the 2003 and 2010 Canadian CFS criteria [4,28], the 2011 International Consensus Criteria on myalgic encephalitis [3], and the Institute of Medicine criteria from 2015 [6]. These criteria require the presence of severe and persistent fatigue over 6 months without medical explanation, accompanied by significant disability and a specific set of symptoms, including headaches, sleep, and cognitive issues, post-exertional malaise, and muscle or joint pain, among others. PCS develops in individuals with either probable or confirmed SARS-CoV-2 infection, which usually emerges about 3 months after initial COVID-19 symptoms and persists for at least 2 months with no alternative diagnosis [29]. Common symptoms include shortness of breath, fatigue, headache, chest and joint pain, and insomnia [29]. The primary review outcome was the severity of fatigue, both physical and mental. Secondary measures included anxiety and depressive symptoms, sleep quality, and overall quality of life. Inflammatory biomarkers and heart rate variability were also considered as outcomes of interest. Clinical trial protocols, editorials, theses dissertations, gray literature, commentaries, conference abstracts, and studies where adults with CFS or PCS were not assessed separately from participants with other conditions were excluded.

### 2.3. Study Selection

Two reviewers (HFC and JSG) independently used Rayyan, a web-based automated screening tool (https://www.rayyan.ai) [30], to conduct the selection process. Duplicate records were manually removed. The remaining studies underwent initial screening by title and abstract, followed by the full text evaluation of eligible records and those without an abstract. Reviewers met with a third reviewer (AMHR) to reach consensus on discrepancies, if needed.

### 2.4. Risk of Bias Assessment

Two independent reviewers (HFC and LFS) evaluated the risk of bias of RCTs with the revised Cochrane Risk of Bias tool version 2 (RoB-2). This tool consists of five domains, with an overall score indicating ‘low’, ‘some concerns’, or ‘high’ risk of bias.

### 2.5. Spin of Information

JSG and LMFS independently evaluated the quality and consistency of information presented in the abstracts of the RCTs. A 7-item checklist was used, with items rated as Yes (presence of spin) or No (absence of spin). This tool evaluates criteria such as the omission of primary outcomes, the selective reporting of positive results, and a failure to mention adverse events [31]. An overall score was calculated [31].

### 2.6. Completeness of Intervention Descriptions

One investigator (HFC) used the Consensus on Exercise Reporting Template (CERT) [32] to check whether qigong-, tai chi-, and/or yoga-based interventions were reported in sufficient detail to allow replicability.

### 2.7. Certainty in the Evidence

The certainty in the evidence was judged by two independent reviewers (AMHR and MJCH) using the Grading of Recommendations, Assessment, Development, and Evaluations (GRADE) approach [31]. The GRADE includes five domains: risk of bias, imprecision, inconsistency, indirectness, and publication bias. RCTs start with high evidence that can be downgraded to moderate, low, or very low [33].

### 2.8. Data Extraction and Synthesis

Two reviewers (HFC and AMHR) independently extracted the following information, whenever possible, using a standardized form: first author, year of publication, and country; number of participants, sex distribution, mean age and body mass index (BMI), and diagnostic criteria; race/ethnicity, education, employment, and financial status; duration of fatigue and associated symptoms; description of the interventions in the mindful exercise and control groups; outcome measures; completion rate, as the number of participants who completed the intervention from those who were initially randomized; main findings; and reported adverse events. Some corresponding authors were contacted to provide or clarify information [34,35,36,37], and all of them responded. The study findings were described narratively. Data from RCTs were collected in an Excel spreadsheet (v.2207), and one researcher (MJCH) pooled results from primary studies to conduct meta-analyses. Mean differences (MD) or standard mean differences (SMD) were calculated using a generic inverse variance method [38]. Fixed or random effects models were used based on the degree of heterogeneity (I^2^ ≥ 50%), with a 95% confidence interval (CI) applied to all analyses. A subgroup analysis explored sources of heterogeneity and examined the impact of mindful exercises and control groups (no intervention/other interventions) on the results. Funnel plots were employed to assess publication bias through asymmetry inspection. Begg’s or Egger’s tests are recommended when at least 10 studies are included in each meta-analysis [38]. Due to the limited number of RCTs, univariate meta-regression analyses were not performed. Review Manager software (RevMan v.5.3, The Cochrane Collaboration, 2020) was used to summarize effects and construct forest plots.

## 3. Results

### 3.1. Study Selection

**Figure 1:**
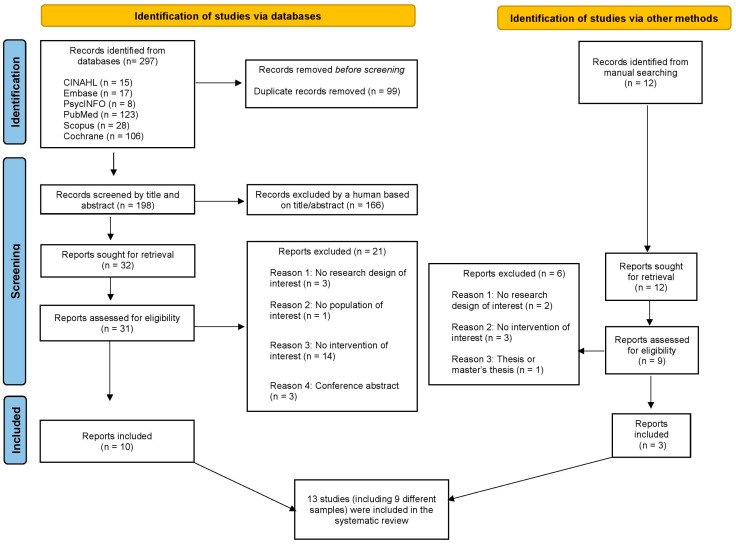
Flow chart diagram of studies through the review process.

A total of 309 relevant records were retrieved from the electronic and manual search strategies. Among these, 13 RCTs (comprising 8 different samples) were included.

(Figure 1). The Supplementary File S2 lists all studies excluded after full-text reading (*n* = 26), along with the reasons for their exclusion.

### 3.2. Description of the Included Studies

The total number of participants was 661 (432 females, 65.35%). Nine RCTs, involving five different samples, evaluated the effect of qigong compared with the waitlist [34,35,39,40,41], usual medical care [42], and cognitive behavioral therapy [43,44,45] in individuals with CFS. The intervention duration ranged from 4 to 17 weeks. Most exercise protocols incorporated 1 or 2 group sessions per week, totaling between 9 and 36 sessions. Various forms of qigong were used, i.e., Baduanjin or Wu Xing Ping Heng Gong, while some studies used the 7- or the 24-form of tai chi. Two clinical trials, including the same sample, explored the impact of an isometric form of yoga versus medical treatment for CFS [36,37]. Additionally, two studies examined the effect of tai chi, one in PCS [46] and the other in CFS [47]. Four studies reported minor and transient adverse events, including dizziness, fatigue, pain, or diarrhea [36,39,43,44]. A comprehensive description of the study characteristics, including exercise protocols in terms of frequency, intensity, time, and type (when available), is provided in Supplementary File S3.

### 3.3. Risk of Bias Assessment

**Figure 2:**
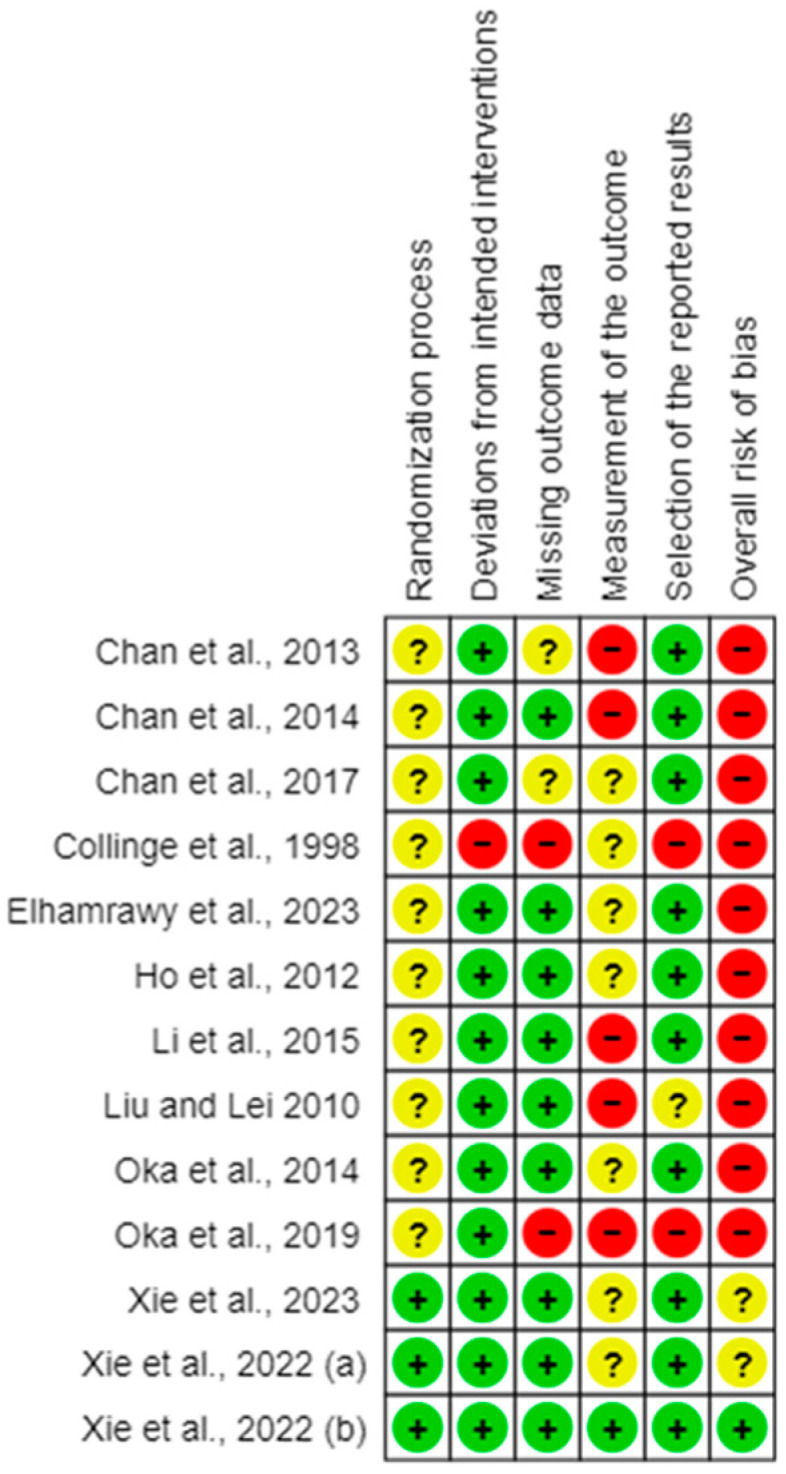
Risk of bias summary. Green circles indicate a low risk of bias; yellow circles with a question mark indicate an unclear risk of bias; red circles indicate a high risk of bias [34,35,36,37,39,40,41,42,43,44,45,46,47].

**Figure 3:**
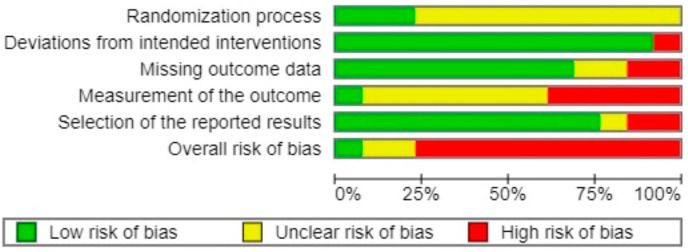
Risk of bias graph.

Only one RCT was considered to have an overall low risk of bias [44] (Figure 2 and Figure 3, inter-rater reliability 62.8%). The most common sources of bias arose from the randomization process, deviations from intended interventions (effect of adhering to intervention), and outcome measurement.

### 3.4. Spin of Information

The overall spin-abstract score was 64, with a mean value of 4.9 points (SD 0.9) (Supplementary File S4; inter-rater reliability, 65%). All abstracts included some form of spin. The most prevalent types were as follows: ‘omission of primary outcomes’ (*n* = 11) and ‘fail to mention adverse events of interventions’ (*n* = 12). In studies where a primary outcome was not identified, items 3 to 7 were scored based on all measures reported in the abstract.

### 3.5. Completeness of Intervention Descriptions

None of the RCTs reported all the items of the CERT checklist. Items 7a and 7b (detailed description of progression or increase in training variables), 15 (rationale to determine at what level participants start the intervention), and 16b (to what extent the intervention was conducted as planned) were absent in all of the studies (Supplementary File S5).

### 3.6. Data Synthesis and Certainty in the Evidence (GRADE)

Six meta-analyses were conducted. Five RCTs were excluded for some meta-analyses due to participants overlap with other studies [35,37,40,41,44]. Using the GRADE approach, serious and very serious concerns were identified regarding risk of bias, inconsistency, indirectness, and the imprecision of the results (Table 1, inter-rater reliability, 90%). The certainty in the evidence was judged as very low for overall fatigue, anxiety, and depressive symptoms, and low for physical and mental fatigue and sleep quality.

#### 3.6.1. Fatigue (GRADE: Low Evidence for Physical and Mental Fatigue and Very Low Evidence for Overall Fatigue)

**Figure 4:**
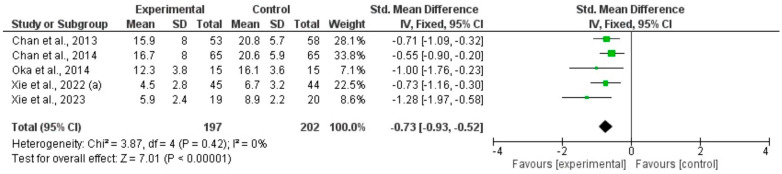
Forest plot of treatment effect for physical fatigue [34,36,39,43,45].

**Figure 5:**
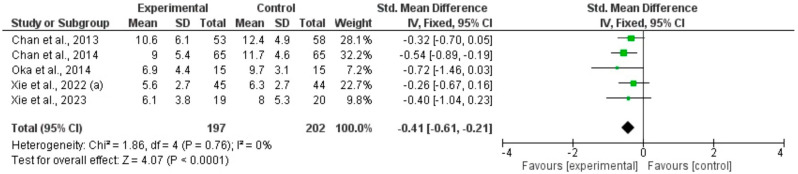
Forest plot of treatment effect for mental fatigue [34,36,39,43,45].

**Figure 6:**
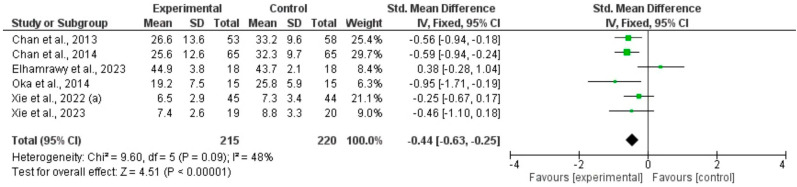
Forest plot of treatment effect for overall fatigue [34,36,39,43,45,46].

Three meta-analyses including six RCTs using qigong, tai chi, or yoga in participants with CFS [34,36,39,43,45] or PCS [46], showed that movement-based mindful exercises significantly reduced the severity of fatigue compared with control interventions, whether physical fatigue: total SMD (95%CI) = −0.73, (−0.93 to −0.52), *p* < 0.0001, I^2^ = 0% (Figure 4), mental fatigue: total SMD (95%CI) = −0.41, (−0.61 to 0.21), *p* < 0.0001, I^2^ = 0%) (Figure 5), or overall fatigue (total: SMD = −0.44, 95% CI [−0.63, −0.25], I^2^ = 48%, *p* < 0.0001) (Figure 6). Subgroup analyses were conducted based on the type of mindful exercise intervention and the control group (Supplementary Files S6–S8). Fatigue symptoms were evaluated with the Chalder Fatigue Scale, the Fatigue Severity Scale, and the Multidimensional Fatigue Inventory. The funnel diagram revealed no publication bias, *p* > 0.05 (Supplementary File S9).

**Table 1:** Certainty in the evidence in the included studies: GRADE.

Summary of Findings			Certainty in the Evidence Based on the GRADE Approach						
Outcome	Studies n (k)	Participants (N/S)	Risk of Bias	Inconsistency	Indirectness	Imprecision	Publication Bias	Level of Evidence	Importance
Anxiety symptoms	4 (4)	360 (360)	−2: Very serious ^a^	No	No	−1: Serious ^b^	No	Very low	Critical
Depressive symptoms	4 (4)	360 (360)	−2: Very serious ^a^	No	No	−1: Serious ^b^	No	Very low	Critical
Fatigue	6 (6)	435 (435)	−2: Very serious ^a^	−1: Serious ^c^	−1: Serious ^d^	No	No	Very low	Critical
Physical fatigue	5 (5)	399 (399)	−2: Very serious ^a^	No	No	No	No	Low	Critical
Mental fatigue	5 (5)	399 (399)	−2: Very serious ^a^	No	No	No	No	Low	Critical
Sleep quality	3 (3)	258 (258)	−1: Serious ^e^	No	No	−1: Serious ^b^	No	Low	Critical

Note: GRADE = Grading of Recommendations Assessment, Development, and Evaluation. N: sample of primary studies in meta-analysis not considering the multi-arm; n: number of studies; k: number of arms of studies included in meta-analysis; S: complete sample included in the meta-analysis considering all multi-arms. ^a^ Risk of bias: two levels were downgraded if at least 50% of the included studies reported a high risk of bias. ^b^ Imprecision: one level was downgraded if the sample size was significantly less than 400 participants. ^c^ Inconsistency: one level was downgraded if moderate heterogeneity was detected (I^2^ = 30–60%). ^d^ Indirectness: one level was downgraded if subgroup differences were detected. ^e^ Risk of bias: one level was downgraded if at least 50% of the included studies reported some concerns of bias.

#### 3.6.2. Anxiety Symptoms (GRADE: Very Low Evidence)

A meta-analysis comprising four RCTs using qigong [34,39,43] or yoga [37] in adults with CFS [34,37,39,43] demonstrated that movement-based mindful exercises were more effective than control interventions in reducing anxiety symptoms: total MD (95%CI) = −1.36, (−2.06 to −0.66), *p* = 0.0001, I^2^ = 14% (Supplementary File S10). Subgroup analyses were conducted based on the type of intervention and the control group (Supplementary File S11). All studies used the Hospital Anxiety and Depression Scale. The funnel diagram showed no publication bias, *p* > 0.05 (Supplementary File S12).

#### 3.6.3. Depressive Symptoms (GRADE: Very Low Evidence)

Four RCTs involving qigong [34,39,43] or yoga [37] in individuals with CFS showed that mindful exercises significantly decreased depressive symptoms compared with control interventions: total MD (95%CI) = −1.94 (−2.67 to) −1.22), *p* < 0.0001, I^2^ = 12% (Supplementary File S13). Subgroup analyses were conducted based on the type of intervention and the control group (Supplementary File S14). Depressive symptoms were assessed with the Hospital Anxiety and Depression Scale. There was no publication bias, *p* > 0.05 (Supplementary File S15).

#### 3.6.4. Quality of Sleep (GRADE: Low Evidence)

A meta-analysis including three RCTs using qigong in patients with CFS [39,43,45] found that qigong was more effective than control interventions to improve sleep quality: total MD (95%CI) = −1.20 (−1.90 to −0.51), *p* = 0.0007, I^2^ = 0%) (Supplementary File S16). A subgroup analysis was conducted based on the control group (Supplementary File S17). All studies used the Pittsburgh Sleep Quality Index. The funnel diagram showed no publication bias, *p* > 0.05 (Supplementary File S18).

## 4. Discussion

This systematic review aimed to summarize and comprehend the efficacy of qigong, tai chi, and yoga to alleviate fatigue and its associated symptoms in adults diagnosed with CFS or PCS. Our meta-analysis involved 13 trials, comprising 8 different samples and a total of 661 participants. The findings suggest that mindful exercises were more effective than control interventions, whether there was no intervention or other interventions (usual medical care or cognitive behavioral therapy), to reduce fatigue and improve anxiety and depressive symptoms and sleep quality. A single RCT used a different form of exercise (aerobic training) as a comparison group [46], which prevents the conclusive determination of whether mindful exercises provide similar benefits than other forms of exercise in this population [16]. Additionally, we only included one study involving participants with PCS [46]. This may not be surprising due to the somehow recent emergence of this syndrome. Given the similarities between CFS and PCS, leveraging available evidence on the effects of exercise therapy in CFS can facilitate the development of evidence-based strategies in clinical practice, while contributing to a broader body of scientific literature for PCS.

Our results are in line with previous reviews on qigong [25] and tai chi [27] for the treatment of fatigue in different conditions and those recently published by Kong et al. in CFS [26]. There are, however, notable differences in our content and analysis. In this review, we made a clear distinction between physical and mental fatigue. Fatigue is recognized as a multidimensional construct comprising both physical and mental components. Physical fatigue refers to challenges in physical activities, whereas mental fatigue pertains to difficulties in cognitive tasks. This differentiation is crucial given the high prevalence of various types of fatigue that can occur simultaneously or separately in people with chronic diseases [48]. Additionally, we incorporated updated evidence on qigong [44,45] and included RCTs using yoga [36,37], since yoga has been demonstrated to alleviate fatigue associated with other conditions, e.g., multiple sclerosis or cancer [49,50]. Yoga has become the most popular form of mindful exercise in some Western cultures [23], where the emphasis has shifted towards its physical aspect as a low-intensity exercise, far from its origins as a spiritual practice [51]. Subgroup analyses showed low heterogeneity, considering the intervention and comparison group (no intervention/other interventions), which indicates similar effects regardless of the type of movement-based mindful exercise. The positive results on anxiety, depression, and quality of sleep also align with former reviews in patients with CFS [26] or cancer [52]. Due to the small number of RCTs involving yoga or tai chi, more clinical studies using these forms of exercise are warranted.

A common criticism in this field is the heterogeneity and poor quality of intervention protocols [53,54,55]. For example, Katla et al. [53] recently recommended improving the quality of yoga-based interventions and developed a checklist for assessment [53]. Using such tools, along with the CERT principles, is necessary to enhance the replicability of the primary studies. Variability in intervention duration, sessions length, and frequency make it difficult to establish a standardized dosage [54,55]. According to the previous literature, recommendations have been set for a minimum duration of one [56] to three months [57], with sessions lasting from 20 min [56] to 60 min [58]. Most evidence indicates that a frequency of two to three sessions per week is necessary to achieve meaningful effects [56,57,58], although these recommendations are based on highly heterogeneous studies. In our review, movement-based mindful interventions averaged 11 weeks in duration (a minimum of 4 weeks), a session length of approximately 75 min (a minimum of 20 min), and a frequency of two sessions per week. Overall, all of these parameters seem to align with existing recommendations.

The safety of interventions needs to be ensured in clinical research, especially when addressing exercise therapy and chronic conditions characterized by fatigue and pain, such as CFS and PCS [59,60]. Exercise practice is commonly viewed as harmless, which may explain why some clinical trials may not even collect potential adverse events. Individuals with CFS or PCS can respond adversely to physical and mental efforts, with not enough evidence supporting the safety of graded exercise therapy (GET) [16]. Indeed, GET has been suggested to elicit large negative responses in over half of CFS patients [61]. The barrier between a tolerable or intolerable exercise dose is often narrow, and exercise quantification must be very precise [62]. One of the largest RCT on exercise therapy in CFS was the PACE trial [63]. Despite its good results, this study has been a matter of controversy and criticism, including the inadequate reporting of all adverse effects [59,64]. In this review, minor and transient adverse events were reported in four studies [36,39,43,44]. Pacing has been advocated as the best strategy to mitigate negative effects following exercise [62], but more clinical trials should be conducted to better quantify and objectify pacing [65]. Mindful exercises can serve as an effective means to facilitate patient adherence to pacing recommendations, even for patients with low exercise tolerance [66]. Internal focus and mental awareness on bodily perceptions during exercise can enhance an individual’s ability to discern the level of effort and fatigue. Additionally, the low-intensity nature of these exercises highlights their positive role in the early stages of the rehabilitation process.

### Clinical and Methodological Considerations

Following previous recommendations [60], we were stringent when establishing the population eligibility criteria, thus only RCTs with accepted guidelines to diagnose CFS [2,3,4,6,28] or PCS [29] patients were included. Our search syntax did not include the term ‘mindfulness’ or related terms such as ‘mindfulness exercise’, as mindfulness is classified as a cognitive therapy under the psychiatry and psychology category in MeSH terminology. While this may have led to the exclusion of potential studies of interest, our focus was on exercise interventions that combine physical activity with increased awareness. Despite the previous literature on mindful exercises in the treatment of fatigue, specific research is necessary in individuals with CFS, as results cannot always be extrapolated from other populations. Improving the reporting of exercise-based interventions is key to advance our understanding on the efficacy of exercise therapy and facilitate its translation into clinical practice. Comprehensive reporting enables replicability, which is essential for tailoring exercise programs to individual needs. Unfortunately, most of the RCTs failed to adhere to the CERT principles when reporting interventions, which detracts from the clinical applicability of our findings. In addition, the low quality of reported information in RCTs may mislead clinical decision-making. The presence of spin was a prevalent issue in most abstracts. Currently, there is a pressing need for greater transparency in the information provided in abstracts [67]. The REPORT guide is a useful tool that facilitates sources of information about effective and transparent trial reporting [68]. Study authors are encouraged to follow these recommendations. When considering the certainty of the evidence using the GRADE framework, serious and very serious concerns emerged in two domains: (a) risk of bias, with only one trial showing a low risk; and (b) imprecision of the results, which could be attributed to low sample sizes. On the contrary, most meta-analyses showed no heterogeneity, except for overall fatigue. This can be explained because the meta-analysis involved the sole study in PCS where aerobic training was used as the control intervention [46]. The lack of follow-up studies makes it difficult to reflect on the possible effects in the medium- and long-term. Finally, a primary and inherent limitation of the review was our inability to access articles and datasets published within Chinese databases. These resources are often restricted in Western countries, thereby limiting our potential to include other studies of interest.

## 5. Conclusions

This study suggests that three popular forms of movement-based mindful exercises, i.e., qigong, tai chi, and yoga, may be effective to reduce fatigue and improve associated symptoms when compared with no treatment, usual care, or other interventions, in adults diagnosed with CFS or PCS. Due to a lack of primary studies with control groups including physical activity interventions, no conclusions can be drawn regarding whether mindful exercises are superior to other forms of exercise in this population. In addition, the present findings are weakened by serious methodological concerns, including the low methodological quality of studies and the low or very low certainty of the evidence, which precludes from establishing sound clinical recommendations.

## Supplementary Materials

The following supporting information can be downloaded at https://www.mdpi.com/article/10.3390/healthcare12202020/s1: Supplementary File S1. Search strategies and results obtained in the different databases; Supplementary File S2. List of references excluded in the last screening, along with reasons (*n* = 26); Supplementary File S3. Characteristics of included studies; Supplementary File S4. Presence of spin of information in the abstracts of included trials; Supplementary File S5. Consensus on Exercise Reporting Template (CERT) of included clinical trials; Supplementary File S6. Subgroup meta-analyses and forest plots for physical fatigue; Supplementary File S7. Subgroup meta-analyses and forest plots for mental fatigue; Supplementary File S8. Subgroup meta-analyses and forest plots for overall fatigue; Supplementary File S9. Funnel diagrams for physical, mental and overall fatigue; Supplementary File S10. Meta-analysis and forest plot for anxiety symptoms; Supplementary File S11. Subgroup meta-analyses and forest plots for anxiety symptoms; Supplementary File S12. Funnel diagram for anxiety symptoms; Supplementary File S13. Meta-analysis and forest plot for depressive symptoms; Supplementary File S14. Subgroup meta-analyses and forest plots for depressive symptoms; Supplementary File S15. Funnel diagram for depressive symptoms; Supplementary File S16. Meta-analysis and forest plot for quality of sleep; Supplementary File S17. Subgroup meta-analysis and forest plot for quality of sleep; Supplementary File S18. Funnel diagram for quality of sleep.
