# Supplementary material for: Exploring the Effects of Qigong, Tai Chi, and Yoga on Fatigue, Mental Health, and Sleep Quality in Chronic Fatigue and Post-COVID Syndromes: A Systematic Review with Meta-Analysis
Source: Healthcare (Basel). 2024 Oct 11;12(20):2020. doi: 10.3390/healthcare12202020 (PMC11507473; doi:10.3390/healthcare12202020)

Supplementary Files

Supplementary File S1. Search strategies and results obtained in the different data bases.

Searches realized on day 03/10/2023.

Pubmed:

((Post infection syndromes [Mesh] OR chronic fatigue syndrome [Mesh] OR Myalgic Encephalomyelitis [Mesh] OR Long covid [Mesh] OR Post covid condition [Mesh] OR Postviral Fatigue Syndrome [Mesh]) AND (qigong [tiab] OR qi gong [tiab] OR Tai [tiab] OR T'ai [tiab] OR Taiji [tiab] OR Yoga [tiab] OR pranayama [tiab] OR chi kung [tiab] OR Baduanjin [tiab] OR Wuqinxi [tiab] OR Liuzijue [tiab] OR Yijinjing [tiab] OR Exercise [tiab] OR training [tiab] OR Pilates [tiab] OR breathing exercises [tiab])) AND (trial[Title/Abstract])

123 results

Cinahl:

AB (Post infection syndromes OR Chronic Fatigue Syndrome OR Myalgic Encephalomyelitis OR CFS/ME OR Long Covid OR Post Covid Syndrome OR Postviral Fatigue Syndrome) AND AB (qigong OR qi gong OR Tai OR T'ai OR Taiji OR Yoga OR pranayama OR chi kung OR Baduanjin OR Wuqinxi OR Liuzijue OR Yijinjing) AND AB (trial)

15 results

Embase:

('chronic fatigue syndrome'/exp OR 'myalgic encephalomyelitis':ti,ab OR 'long covid'/exp OR 'postviral fatigue syndrome'/exp) AND ('qigong':ti,ab OR 'qi gong':ti,ab OR 'tai':ti,ab OR 't ai':ti,ab OR 'taiji':ti,ab OR 'yoga':ti,ab OR 'pranayama':ti,ab OR 'chi kung':ti,ab OR 'baduanjin':ti,ab OR 'wuqinxi':ti,ab OR 'liuzijue':ti,ab OR 'yijinjing':ti,ab) AND 'trial':ti,ab

17 results

psycINFO:

tiab(Post infection syndromes OR chronic fatigue syndrome OR Myalgic Encephalomyelitis OR CFS/ME OR Long covid OR Post covid syndrome OR Postviral Fatigue Syndrome) AND tiab(qigong OR “qi gong” OR Tai OR T'ai OR Taiji OR Yoga OR pranayama OR “chi kung” OR Baduanjin OR Wuqinxi OR Liuzijue OR Yijinjing) AND tiab(trial)

8 results

Cochrane library:

#1 MeSH descriptor: [Post-Infectious Disorders] explode all trees

#2 MeSH descriptor: [Fatigue Syndrome, Chronic] explode all trees

#3 MeSH descriptor: [Post-Acute COVID-19 Syndrome] explode all trees

#4 (qigong):ti, ab,kw

#5 (qi gong):ti, ab, kw

#6 (Tai):ti,ab,kw

#7 (Taiji):ti, ab,kw

#8 (Yoga):ti,ab,kw

#9 (pranayama):ti,ab,kw

#10 (chi kung):ti,ab,kw

#11 (Baduanjin):ti,ab,kw

#12 (Wuqinxi):ti,ab,kw

#13 (Liuzijue):ti,ab,kw

#14 (Yijinjing):ti,ab,kw

#15 (Exercise):ti,ab,kw

#16 (training):ti,ab,kw

#17 (Pilates):ti,ab,kw

#18 (breathing exercises):ti,ab,kw

#19 (trial):ti,ab,kw

#20 (#1 OR #2 OR #3) AND (#4 OR #5 OR #6 OR #7 OR #8 OR #9 OR #10 OR #11 OR #12 OR #13 OR #14 OR #15 OR #16 OR #17 OR #18)) AND #19

108 results / 106 Clinical Trials

Scopus:

(ABS ("Post infection syndromes" OR "chronic fatigue syndrome" OR "Myalgic Encephalomyelitis" OR "CFS/ME" OR "Long covid" OR "Post covid syndrome" OR "Postviral Fatigue Syndrome")) AND (ABS (qigong OR "qi gong" OR tai OR t'ai OR taiji OR yoga OR pranayama OR "chi kung" OR baduanjin OR wuqinxi OR liuzijue OR yijinjing)) AND (TITLE-ABS-KEY (trial))

28 results

TOTAL RESULTS: 297

DUPLICATES FOUND AUTOMATICALLY: 92

DUPLICATES FOUND BY MANUAL SEARCHING: 7

TOTAL RESULTS WITHOUT DUPLICATES: 198

Supplementary File S2. List of references that were excluded in the last screening, along with reasons (n = 26).

From the systematic search (n = 21):

**Reason 1: No research design of interest (n = 3)**	
1.	Chan JSM, Ng SM, Yuen LP, Chan CLW. Qigong exercise for chronic fatigue syndrome. International Review of Neurobiology [Internet]. 2019. p. 121-53. Available in: https://www.scopus.com/inward/record.uri?eid=2-s2.0-85072968543&doi=10.1016%2fbs.irn.2019.08.002&partnerID=40&md5=0079d9626106bcd9bb0029177eac9099
2.	Reid S, Chalder T, Cleare A, Hotopf M, Wessely S. Chronic fatigue syndrome. BMJ (Clinical research ed). 2000;320(7230):292-6.
3.	Wu K, Li Y, Zou Y, Ren Y, Wang Y, Hu X, et al. Tai Chi increases functional connectivity and decreases chronic fatigue syndrome: A pilot intervention study with machine learning and fMRI analysis. PLoS One. 2022;17(12):e0278415.
**Reason 2: No population of interest (n = 1)**	
1.	Ridsdale L, Darbishire L, Seed PT. Is graded exercise better than cognitive behaviour therapy for fatigue? A UK randomized trial in primary care. Psychol Med. 2004;34(1):37-49.
**Reason 3: No intervention of interest (n = 14)**	
1.	Besnier F, Bérubé B, Malo J, Gagnon C, Grégoire CA, Juneau M, et al. Cardiopulmonary Rehabilitation in Long-COVID-19 Patients with Persistent Breathlessness and Fatigue: The COVID-Rehab Study. Int J Environ Res Public Health. 2022;19(7).

2.	Clark LV, Pesola F, Thomas JM, Vergara-Williamson M, Beynon M, White PD. Guided graded exercise self-help plus specialist medical care versus specialist medical care alone for chronic fatigue syndrome (GETSET): a pragmatic randomised controlled trial. Lancet. 2017;390(10092):363-73.
3.	Estebanez-Pérez MJ, Pastora-Bernal JM, Martín-Valero R. The Effectiveness of a Four-Week Digital Physiotherapy Intervention to Improve Functional Capacity and Adherence to Intervention in Patients with Long COVID-19. Int J Environ Res Public Health. 2022;19(15).
4.	Fernie BA, Murphy G, Wells A, Nikčević AV, Spada MM. Treatment Outcome and Metacognitive Change in CBT and GET for Chronic Fatigue Syndrome. Behav Cogn Psychother. 2016;44(4):397-409.
5.	Fulcher KY, White PD. Randomised controlled trial of graded exercise in patients with the chronic fatigue syndrome. BMJ. 1997;314(7095):1647-52.
6.	McNarry MA, Berg RMG, Shelley J, Hudson J, Saynor ZL, Duckers J, et al. Inspiratory muscle training enhances recovery post-COVID-19: a randomised controlled trial. Eur Respir J. 2022;60(4).
7.	Moss-Morris R, Sharon C, Tobin R, Baldi JC. A randomized controlled graded exercise trial for chronic fatigue syndrome: outcomes and mechanisms of change. J Health Psychol. 2005;10(2):245-59.
8.	Núñez M, Fernández-Solà J, Nuñez E, Fernández-Huerta JM, Godás-Sieso T, Gomez-Gil E. Health-related quality of life in patients with chronic fatigue syndrome: group cognitive behavioural therapy and graded exercise versus usual treatment. A randomised controlled trial with 1 year of follow-up. Clin Rheumatol. 2011;30(3):381-9.

9.	Palau P, Domínguez E, Gonzalez C, Bondía E, Albiach C, Sastre C, et al. Effect of a home-based inspiratory muscle training programme on functional capacity in postdischarged patients with long COVID: the InsCOVID trial. BMJ Open Respir Res. 2022;9(1).
10.	Ridsdale L, Hurley M, King M, McCrone P, Donaldson N. The effect of counselling, graded exercise and usual care for people with chronic fatigue in primary care: a randomized trial. Psychol Med. 2012;42(10):2217-24.
11.	Romanet C, Wormser J, Fels A, Lucas P, Prudat C, Sacco E, et al. Effectiveness of exercise training on the dyspnoea of individuals with long COVID: A randomised controlled multicentre trial. Ann Phys Rehabil Med. 2023;66(5):101765.
12.	Sabes-Figuera R, McCrone P, Hurley M, King M, Donaldson AN, Ridsdale L. Cost-effectiveness of counselling, graded-exercise and usual care for chronic fatigue: evidence from a randomised trial in primary care. BMC Health Serv Res. 2012;12:264.
13.	Wallman KE, Morton AR, Goodman C, Grove R, Guilfoyle AM. Randomised controlled trial of graded exercise in chronic fatigue syndrome. Med J Aust. 2004;180(9):444-8.
14.	Windthorst P, Mazurak N, Kuske M, Hipp A, Giel KE, Enck P, et al. Heart rate variability biofeedback therapy and graded exercise training in management of chronic fatigue syndrome: An exploratory pilot study. J Psychosom Res. 2017;93:6-13.
**Reason 4: Conference abstract (n = 3)**	
1.	Oka T, Yamada Y. Recumbent Isometric Yoga Alters Circulating Microrna in Patients with Myalgic Encephalomyelitis/Chronic Fatigue Syndrome. Psychosom Med. 2020;82(6):A94.

2.	Oka T, Yamada Y. Effects of recumbent isometric yoga on patients with myalgic encephalpmyelitis/chronic fatigue syndrome: A randomized, controlled trial. Psychosom Med. 2019;81(4):A97.
3.	Oka T, Tanahashi T, Sudo N. Effect of isometric yoga on chronic fatigue syndrome: A randomized controlled trial. Psychother Psychosom. 2013;82:78-9.

From the manual search (n= 5):

**Reason 1. The intervention is not of interest. (n = 2)**	
1.	Sollie, K.; Næss, E.T.; Solhaug, I.; Thimm, J. Mindfulness training for chronic fatigue syndrome: A pilot study. Health Psychol. Rep. 2017, 3, 240–250.
2.	Surawy, C.; Roberts, J.; Silver, A. The Effect of Mindfulness Training on Mood and Measures of Fatigue, Activity, and Quality of Life in Patients with Chronic Fatigue Syndrome on a Hospital Waiting List: A Series of Exploratory Studies. Behav. Cogn. Psychother. 2005, 33, 103–109.
3.	Rimes, K.A.; Wingrove, J. Mindfulness-Based Cognitive Therapy for People with Chronic Fatigue Syndrome Still Experiencing Excessive Fatigue after Cognitive Behaviour Therapy: A Pilot Randomized Study. Clin. Psychol. Psychother. 2013, 20, 107–117.
**Reason 2. The research design in not of interest (n = 2)**	
4.	Oka, T.; Tanahashi, T.; Sudo, N.; Lkhagvasuren, B.; Yamada, Y. Changes in fatigue, autonomic functions, and blood biomarkers due to sitting isometric yoga in patients with chronic fatigue syndrome. BioPsychoSocial Med. 2018, 12, 3.

5.	Takakura, S.; Oka, T.; Sudo, N. Changes in circulating microRNA after recumbent isometric yoga practice by patients with myalgic encephalomyelitis/chronic fatigue syndrome: An explorative pilot study. Bio. Psycho. Soc. Med. 2019, 13, 1–10.
**Reason 3: Thesis and master's thesis (n = 1)**	
6.	Dybwad MH, Frøslie KF, Stanghelle JK: Work capacity, fatigue and health related quality of life in patients with myalgic encephalopathy or chronic fatigue syndrome, before and after qigong Therapy, a randomized controlled study. Nesoddtangen, Norway: Sunnaas Rehabilitation Hospital 2007 [http://old.sunnaas.no/stream_file.asp? iEntityId=7623].

Supplementary File S3. Characteristics of included studies.

Author(s), year; and country	Participants (sex), age, groups. BMI Diagnostic criteria	Race/ethnicity<br>Education<br>Employment<br>Family / financial status	Fatigue duration. Other symptoms	Intervention, mindful exercise group: duration	Comparison group: duration	Outcomes of interest. Assessment points	Completion rate[a]	Main findings<br>Adverse events
**Qigong**								
Chan et al. 2013; China	N=137 (105 females) [b]: QG=72, CG=65. Mean age (SD), QG: 42.4 (6.7), CG: 42.5 (6.4) BMI: UR CFS-like illness (self-reported): 1994 US CDC Diagnostic Criteria	Race/ethnicity: Chinese. Education: secondary school (n=64); tertiary or above (n=73). Employment: full-time (n=107); part-time (n=4); housewife (n=19); unemployed (n=5); other (n=2). Family status: single (n=44); married/cohabiting (n=84); divorced/separated/widowed (n=9) Financial status (monthly income, HKD): <10,000 (n=17); 10,000-19,999 (n=38); 20,000-29,999	Fatigue duration: UR Other symptoms (over 24 wks.): poor sleep (n=129); muscle pain (n=128); impaired memory or concentration (n=126)	Qigong (Wu Xing Ping Heng Gong, 10 form): 10 sessions, twice weekly, 2 hrs. each: 45 min. theory, 15 min. meditation, and 1hr. Qigong exercise training. Qigong self-practice (at least 30 min. daily), 12 wks. Total: 17 wks.	Waitlist: lifestyle as usual, 17 wks.	Anxiety symptoms: HADS Depressive symptoms: HADS Fatigue (physical and mental): ChFS T0, T1 (week 17)	Overall: 72.1% (111/154) QG: 68.8% (53/77) CG: 75.3% (58/77)	Significant differences between groups for fatigue and depressive symptoms.<br>AE: None reported

		(n=17); ≥30,000 (n=19); no income/not available (n=17); no answer (n=29)						
Chan et al. 2014; China	N=150 (108 females): QG=75, CG=75. Mean age (SD), QG: 39.1 (7.8), CG: 38.9 (8.1) BMI (mean, SD): QG: 22.3 (4.9), CG: 21.6 (3.4) CFS-like illness (self-reported): 1994 US CDC Diagnostic Criteria	Race/ethnicity: Chinese. Education: secondary or below (n=56); tertiary or undergraduate (n=66); master or above (n=28). Employment: full time (n=135); part time (n=4); housewife/ unemployed/ retired (n=11). Family status: single (n=55); married/cohabiting (n= 99); divorced/separated (n=6). Financial status (household monthly income, HKD): <10,000 (n=21); 10,000-19,999 (n=44); 20,000-29,999 (n=29); ≥30,000 (n=28); no income (n=7); no answer (n=21)	Fatigue duration: 1-2 yrs. (n=61); 2-5 yrs. (n=56); >5 yrs. (n=33) Other symptoms (over 24 wks.): poor sleep (n=144), muscle pain (n=143), impaired memory / concentration (n=132), post effort malaise (n=106), joint pain (n=84), headache (n=82), tender lymph nodes (n=62), sore throat (n=55)	Qigong (Baduanjin Qigong, 8 standard movements): 16 group sessions, 90 min. each: 15 min. relaxation; 25 min. explanation, 20 min. big group Qigong practice, and 30 min. small group practice. Qigong self-practice (at least 30 min. daily). Total: 9 wks.	Waitlist: lifestyle as usual, 9 wks.	Anxiety symptoms: HADS Depressive symptoms: HADS Fatigue (physical and mental): ChFS Sleep quality: PSQI T0, T1 (week 9), T2 (week 21)	Overall: 86.6% (130/150) QG: 86.6% (65/75) CG: 86.6% (65/75)	Significant differences between groups for all outcomes (HADS - depression, and PSQI only at T1). AE: muscle ache (n=24), palpitation (n=4), giddiness (n=3), knee, back, or shoulder pain (n=5), dizziness (n=2), fatigue (n=2), nervousness (n=2), Qi movement inside body (n=2), tight

								chest (n=1), breathlessness (n=1), sleepier (n=1)
Chan et al. 2017; China	N= 108 (108 females) [c]: QG= 46, CG= 62. Median age (IQ range), QG: 39.5 (33.5-45.3) CG: 42 (32.5-47) BMI (median, IQ): QG: 20.8 (18-23.7) CG: 20.1 (19.1-22.9) CFS-like illness (self-reported): 1994 US CDC	Race/ethnicity: Chinese. Education: secondary or below (n=45); tertiary/undergraduate or above (n=63). Employment: full time (n=93); others (n= 15). Family status: single/divorced/ separated (n= 45); married/cohabiting (n= 63). Financial status: UR	Fatigue duration: UR Other symptoms: UR	Qigong (Baduanjin Qigong, 8 standard movements): 16 group sessions, 90 min. each Qigong self-practice (at least 30 min. daily). Total: 9 wks.	Waitlist: lifestyle as usual, 9 wks.	Anxiety symptoms: HADS Blood marker: Plasma adiponectin level Depressive symptoms: HADS T0, T1 (week 9), T2 (week 21)	Overall: UR QG: UR CG: UR	Significant differences between groups for all outcomes only at T1. AE: UR

	Diagnostic Criteria							
Collinge et al. 1998; US	N= 70 (50 females) [b]: QG=37, CG=33. Mean age (SD), sample: 44.2 (7.0) BMI: UR CFS (physician-diagnosed): 1994 US CDC Diagnostic Criteria	Race/ethnicity: Caucasian (n= 55); Hispanic (n=3); Native American (n=1); Asian American (n=1). Education: high school (n=2); some college (n=18); bachelor (n=26); master's or doctorate (n=14). Employment: homemaker (n=2); student (n=1); employee (n=57). Family status: single (n=35); living with spouse or partner (n= 21); separated (n=4). Financial status: UR	Fatigue duration: mean (SD), 4.75 (2.3) yrs., range 1-11.9 yrs. Other symptoms: UR	Behavioral program: 9 group sessions, 2 hrs. each: medical Qigong (30 min.), mindfulness meditation (30 min.), group discussion (1hr.). Self-practice (at least 30 min. daily). Total: 9 wks.	Usual medical care, 9 wks.	Health-related QoL: SF-36 Symptoms severity: SIS T0, T1 (week 10), T2 (week 52)	Overall: 85% (60/70) QG: 75% (28/37) CG: 96% (32/33)	No data reported for differences within or between groups. AE: UR
Ho et al. 2012; China	N=70 (51 females) [b,d]: QG= 35, CG= 35. Mean age (SD), QG: 42.1 (7.3),	Race/ethnicity: UR Education: secondary or high school (n=29); college or above (n=35).	Fatigue duration: UR Other symptoms: impaired memory or concentration (n=60), poor sleep (n=62),	Qigong (Wu Xing Ping Heng Gong, 10 form): 10 sessions, twice weekly, 2 hrs. each: 30-40 min. theory,	Waitlist: lifestyle as usual, 17 wks.	Fatigue (physical and mental): ChFS Health-related QoL (physical and mental):	Overall: 74.3% (52/70) QG: 77.1% (27/35)	Significant differences between groups for fatigue and SF-12 (mental). Differences in

	CG: 42.5 (5.5)<br>BMI: UR<br>CFS: 1994 US CDC Diagnostic Criteria	Employment: full time (n=51); part time (n=2); housewife (n=9); unemployed (n= 2).<br>Family status: single (n=20); married / cohabited (n=38); divorced / separated (n=6).<br>Financial status: UR	muscle pain (n=58), joint pain (n=47), post-effort malaise (n=58), headache (n=44), sore throat (n=35), tender lymph nodes (n=45)	20 min. meditation, and 1hr. Qigong exercise training. Qigong self-practice (at least 30 min. daily), 12 wks.<br>Total: 17 wks.		SF-12<br>Blood marker: Telomerase activity<br>T0, T1 (week 5), T2 (week 17)	CG: 71.4% (25/35)	telomerase activity were reported at T1.<br>AE: None reported
Li et al. 2015; China	N= 46 (40 females) [d]: QG=22, CG=24.<br>Median age (range),<br>QG: 46 (23–52),<br>CG: 45 (32–51).<br>BMI: UR<br>CFS-like illness (self-reported): 1994 US CDC	Race/ethnicity: Chinese.<br>Education: secondary or below (n=27); tertiary / university (n= 15); master's level or above (n=4).<br>Employment: full time (n= 36); part time (n=2); housewife (n=4); unemployed (n=2); other (n=2).<br>Family status: single (n=10); married / cohabited (n= 30); divorced / separated (n=6).<br>Financial status: UR	Fatigue duration: UR<br>Other symptoms: UR	Qigong (Wu Xing Ping Heng Gong, 10 form): 10 sessions, twice weekly, 2 hrs. each: 45 min. theory, 15 min. warm-up, and 1hr. Qigong exercise training. Qigong self-practice (15-30 min. at least 3 times per week), 12 wks.<br>Total: 17 wks.	Waitlist: lifestyle as usual, 17 wks.	Anxiety symptoms: HADS<br>Depressive symptoms: HADS<br>Fatigue (physical and mental): ChFS<br>Health-related QoL (physical and mental): SF-12<br>T0, T1 (week 17)	Overall: 100% (46/46)<br>QG: 100% (22/22)<br>CG: 100% (24/24)	Significant differences between groups for fatigue (physical and mental) and SF-12 (mental)<br>AE: None reported

	Diagnostic Criteria							
Xie et al. 2022a; China	N=90 (53 females) [b]: QG=45, CG=45. Mean age (SD), QG: 37.9 (11.3), CG: 37.3 (9.8) BMI (mean): QG: 22.4, CG: 22.8 CFS-like illness (self-reported or physician diagnosed): 1994 US CDC Diagnostic Criteria	Race/ethnicity: UR Education, mean yrs. (SD): QG, 11.8 (3.2); CG, 11.2 (2.9) Employment: UR. Family status: single (n=45); married (n=41); divorced (n=3). Financial status: UR	Fatigue duration: UR Other symptoms: UR	Qigong (PLWNT): 1 group session per week (1 h. each): 10 min. relaxation, 20 min. guidance, and 30 min. group practice. Qigong self-practice (30 min. daily, 6 days per week). Total: 12 wks.	CBT: 1 group session per week (1h each). Home listening of lectures (30 min. daily, 6 days per week), 12 wks.	Anxiety symptoms: HADS Blood marker: NPY Depressive symptoms: HADS Fatigue (physical and mental): MFI-20 Sleep quality: PSQI T0, T1 (week 12)	Overall: 98% (89/90) QG: 100% (45/45) CG: 97% (44/45)	No differences between groups for any variable, except NPY. AE: leg, chest, or thumb pain (n=3), dizziness (n=1), diarrhea (n=1)

Xie et al. 2022b; China	N=34 (17 females) [b, e]: QG=15, CG=15. Mean age (SD), QG: 37.9 (11.3), CG: 37.3 (9.9) BMI (mean): QG: 22.4, CG: 22.8 CFS-like illness (self-reported or physician diagnosed): 1994 US CDC Diagnostic Criteria	Race/ethnicity: UR Education, mean yrs. (SD): QG, 11.8 (3.2); CG, 11.2 (2.9) Employment: UR Family status: UR Financial status: UR	Fatigue duration: UR Other symptoms: UR	Qigong (PLWNT): 1 group session per week (1 h. each): 10 min. relaxation, 30 min. guidance, and 20 min. group practice. Qigong self-practice (30 min. daily, 6 days per week). Total: 12 wks.	CBT: 1 group session per week (1h each). Home listening of lectures (30 min. daily, 6 days per week), 12 wks.	Fatigue (physical and mental): MFI-20 Health-related QoL (global): SF-36 T0, T1 (week 12)	Overall: 88% (30/34) QG: 88% (15/17) CG: 88% (15/17)	Significant differences between groups for SF-36. AE: thumb pain (n=1)
Xie et al. 2023; China	N= 40 (27 females) [b]: QG=19, CG=20.	Race/ethnicity: UR. Education: UR Employment: UR	Fatigue duration: UR Other symptoms: UR	Qigong (Yijinjing, 12 movements): 1 group session per week (1h.	CBT: 1 group session per week (1h	Fatigue (physical and mental): MFI-20	Overall: 97% (39/40)	Significant differences between groups for all

	Mean age (SD), QG: 35.1 (6.1), CG: 36.7 (8.4) BMI: UR CFS (self-reported): 1994 US CDC Diagnostic Criteria	Family status: UR Financial status: UR		each): 10 min. relaxation; 30 min. guidance, and 20 min. group practice. Qigong self-practice (30 min. daily, 5 days per week). Total: 12 wks.	each). Home listening of lectures (30 min. daily, 5 days per week), 12 wks.	Health-related QoL (physical and mental): SF-36 Sleep quality: PSQI T0, T1 (week 12)	QG: 95% (19/20) CG: 100% (20/20)	variables except SF-36 (mental). AE: None reported
**Yoga**								
Oka et al. 2014; Japan	N=30 (24 females): YG=15, CG=15. Mean age (SD), YG: 38 (11.1), CG: 39.1 (14.2) BMI: UR	Race/ethnicity: UR Education: UR Employment: UR Family status: UR Financial status: UR	Fatigue duration: UR Other symptoms: UR	Yoga (isometric form, 6 seated poses): 1 individual session every 2 to 3 wks. (20 min each), and self-practice at home (daily, 20 min) + medical treatment. Total (mean, SD): 9,2 (2,5) wks.	Medical treatment Total (mean, SD): 9,2 (2,5) wks.	Fatigue (physical and mental): ChFS Health-related QoL (physical and mental): SF-8 T0, T1 (week 9 approx.)	Overall: 100% (30/30) YG: 100% (15/15) CG: 100% (15/15)	Significant differences between groups for fatigue AE: dizziness (n=1), tiredness (n=2), light-headed (n=2)

	CFS: 1994 US CDC Diagnostic Criteria							
Oka et al. 2019; Japan	N= 30 (24 females) [f]: YG=15, CG=15. Mean age (SD), YG: 38 (11.1), CG: 39.1 (14.2) BMI: UR CFS: 1994 US CDC Diagnostic Criteria	Race/ethnicity: UR Education: UR Employment: UR Family status: UR Financial status: UR	Fatigue duration: UR Other symptoms: UR	Yoga (isometric form, 6 seated poses): 1 individual session every 2 wks. (20 min each), and self-practice at home (daily, 20 min) + medical treatment. Total: approx. 8 wks.	Medical treatment, approx. 8 wks.	Alexithymia: TAS-20 Anxiety symptoms: HADS Depressive symptoms: HADS Fatigue: ChFS Blood markers: cortisol, prolactin, DHEA-S, TGF-β1, TNF-α, carnitine, BDNF, MHPG, HVA, IL-6, α-MSH T0, T1 (week 8)	Overall: 100% (30/30) YG: 100% (15/15) CG: 100% (15/15)	Significant differences between groups only for HADS (depressive symptoms). AE: none reported

**Tai Chi**								
Elhamrawy et al. 2023; Egypt	N= 54 (19 females): TCG=18, ATG=18, CG=18. Mean age (SD), TCG: 65.7 (3.6), ATG: 66.2 (3.8), CG: 66.3 (4) BMI, mean (SD): TCG: 27.4 (1.9), ATG: 28.7 (1.3), CG: 28.1 (1.8) Post-COVID-19: mild to moderate (PCFSS)	Race/ethnicity: UR Education: UR Employment: UR Family status: UR Financial status: UR	Months post-COVID 19, mean (SD): TCG=7.1 (2.3), ATG=7.2 (1.8), CG=7.6 (2.2) Other symptoms: UR	Tai Chi (7 TC movements): 4 sessions per week (60 min. each): 10 min. warm-up, 40 min. TC, and 10 min. relaxation, 12 wks.	ATG: 4 sessions per week (60 min. each): 10 min. stretch, 20 min. strength, treadmill walking 15-20 min., and 10 min. stretch, 12 wks. CG: usual activities	Fatigue: FSS Physical performance: GS, SFT (30-second arm curls and chair stands, 8-FUG, 2-min. step test T0, T1 (week 12)	Overall: 100% (54/54) TCG: 100% (18/18) ATG: 100% (18/18) CG: 100% (18/18)	Significant differences between TCG and ATG vs. CG for all outcomes. TCG was better than ATG for GS and arm curls. AE: none reported
Liu & Lei 2010; China	N=90 (46 females):	Race/ethnicity: UR Education: UR Employment: UR	Fatigue duration: mean (SD), yrs. TG: 2.2 (1.6),	Tai Chi (24 simplified Tai Chi forms): 1 group	CG1: massage (One Finger	Blood markers: MDA,	Overall: 100% (90/90)	No significant differences between

	TCG=30, CG1=30, CG2=30. Mean age (SD), TCG: 35.1 (4.0), CG1: 34.7 (4.1), CG2: 35.4 (3.8) BMI: UR CFS: 1994 US CDC Diagnostic Criteria	Family status: UR Financial status: UR	CG1: 2.3 (1.8) CG2: 2.2 (1.5) Other symptoms: UR	session daily (30 min. each), 4 wks. (30 days)	Zen and Twisting methods), 1 group session every other day (30 min. each), 4 wks. CG2: drugs (Fluoxetine, once daily), 4 wks.	SOD, and GSH-Px. T0, T1 (week 4)	TCG: 100% (30/30) CG1: 100% (30/30) CG2: 100% (30/30)	groups for any variable. AE: none reported

Abbreviations: AE, adverse events; ATG, aerobic training group; BDNF, brain-derived neuro-trophic factor; BMI, body mass index; CDC, Centers for Disease Control and Prevention; CFS, Chronic Fatigue Syndrome; ChFS, Chalder Fatigue Scale; CG, control group; DHEA-S, Dehydroepiandrosterone sulfate; FSS, Fatigue Severity Scale; FUG, Foot up and go; GS, hand grip strength; GSH-Px, serum glutathione peroxidase; HADS, Hospital Anxiety and Depression Scale; HKD, Hong Kong Dollar; hrs, hours; HVA, homovanillic acid; IL-6, Interleukin-6; IQ, interquartile; MDA, malondialdehyde; min., minutes; MFI-20, Multidimensional Fatigue Inventory 20; MHPG, 3-Methoxy-4-hydroxyphenylglycol;; α-MSH, Melanocyte-stimulating hormone alpha; NPY, neuropeptide Y; PCFSS, Post-COVID-19 Functional Status Scale; PLWNT, prolong life with nine turn method; PSQI, Pittsburgh Sleep Quality Index; QG, qigong group; QoL, quality of life; SD, standard deviation; SFT, Senior Fitness Test; SF-12, 12-Item Short-Form Health Survey; SF-36, 36-item Short Form Health Survey; SF-8TM, Medical Outcomes Study Short Form 8; SIS, Symptom Index Survey; SOD, serum superoxide dismutases; T0, baseline; T1, post-intervention; T2, follow-up; TAS-20, 20-item Toronto Alexithymia Scale; TCG, Tai Chi group; TGF-β1, Transforming growth factor beta 1; TNF-α, tumor necrosis factor; UR, unreported; US, United States; wks., weeks; YG, yoga group; yrs., years;

[a] Treatment completion rate refers to the number of participants who completed the intervention from those who were initially randomized.; [b] Participants characteristics are reported from those who started the intervention; [c] Same study sample as Chan et al. 2014; [d] Same study sample as Chan et al. 2013; [e] Same study sample as Xie et al. 2022a; [f] Same study sample as Oka et al. 2014

Adverse events are referred to the movement-based mindful intervention.

Supplementary File S4. Presence of spin of information in the abstracts of included trials (SPIN checklist, n = 13).

Items	Yes<br>n (%)	No<br>n (%)
Item 1: Omission of primary outcomes	11 (84.6%)	2 (15.4%)
Item 2: Fail to mention adverse events of interventions	12 (92.3%)	1 (7.7%)
Item 3: Selective reporting of positive results and omission of negative results of primary outcomes	9 (69.2%)	4 (30.8%)
Item 4: Fail to report statistically nonsignificant primary outcomes	10 (76.9%)	3 (23.1%)
Item 5: Focus on statistically significant outcomes other than the primary	5 (38.5%)	8 (61.5%)
Item 6: Overenthusiastic interpretation of statistically nonsignificant primary outcome results as effective	9 (%)	4 (30.8%)
Item 7: Recommendation of a treatment without a clinically important effect on primary outcomes	8 (61.5%)	5 (38.5%)

Yes, spin is clearly present, the primary outcome results are not reported, or the primary outcome results are omitted; No, spin is not present.

Item 1: Omission of primary outcomes.

Item 2: Fail to mention adverse events of interventions.

Item 3: Selective reporting of positive results and omission of negative results of primary outcomes.

Item 4: Fail to report statistically nonsignificant primary outcomes.

Item 5: Focus on statistically significant outcomes other than the primary.

Item 6: Overenthusiastic interpretation of statistically nonsignificant primary outcome results as effective.

Item 7: Recommendation of a treatment without a clinically important effect on primary outcomes.

Supplementary File S5. Consensus on Exercise Reporting Template (CERT) of included clinical trials.

**Author(s)**	**1**	**2**	**3**	**4**	**5**	**6**	**7a**	**7b**	**8**	**9**	**10**	**11**	**12**	**13**	**14a**	**14b**	**15**	**16a**	**16b**
**QIGONG**																			
Chan et al. 2013; China	No (NM)	Yes	No (NM)	No (NM)	Yes	No (NM)	No (NM)	No (NM)	Yes	Yes	Yes	Yes	No (NM)	No (NPD)	No (NM)	No (NM)	No (NM)	No (NM)	No (NM)
Chan et al. 2014; China	No (NM)	Yes	Yes	Yes	Yes	Yes	No (NM)	No (NM)	Yes	Yes	Yes	Yes	No (NM)	No (NM)	Yes	No (NM)	No (NM)	No (NM)	No (NM)
Chan et al. 2017; China	No (NM)	Yes	No (NPD)	No (NM)	No (NM)	No (NM)	No (NM)	No (NM)	No (NM)	Yes	No (NM)	No (NM)	No (NM)	No (NM)	No (NM)	No (NM)	No (NM)	No (NM)	No (NM)
Collinge et al. 1998; USA	No (NM)	No (NM)	No (NM)	No (NM)	Yes	Yes	No (NM)	No (NM)	No (NM)	Yes	Yes	No (NM)	No (NM)	No (NM)	No (NM)	No (NM)	No (NM)	No (NM)	No (NM)
Ho et al. 2012; China	No (NM)	Yes	No (NM)	No (NM)	Yes	No (NM)	No (NM)	No (NM)	No (NM)	Yes	Yes	Yes	No (NM)	No (NM)	No (NM)	No (NM)	No (NM)	No (NM)	No (NM)
Li et al. 2015; China	No (NM)	Yes	No (NM)	No (NM)	Yes	No (NM)	No (NM)	No (NM)	Yes	Yes	Yes	Yes	No (NM)	No (NPD)	No (NM)	No (NM)	No (NM)	No (NM)	No (NM)
Xie et al (a). 2022; China	No (NM)	Yes	Yes	Yes	Yes	Yes	No (NM)	No (NM)	Yes	Yes	Yes	Yes	Yes	Yes	Yes	No (NPD)	No (NM)	No (NM)	No (NM)
Xie et al (b). 2022; China	No (NM)	Yes	Yes	Yes	Yes	Yes	No (NM)	No (NM)	Yes	Yes	Yes	Yes	Yes	Yes	Yes	No (NPD)	No (NM)	No (NM)	No (NM)

Xie et al. 2023; China	No (NM)	Yes	Yes	Yes	Yes	Yes	No (NM)	No (NM)	Yes	Yes	Yes	Yes	No (NM)	No (NM)	No (NM)	No (NM)	No (NM)	Yes	No (NM)
**YOGA**																			
Oka et al. 2014; Japan	Yes	Yes	Yes	Yes	Yes	Yes	No (NM)	No (NPD)	Yes	Yes	Yes	Yes	Yes	Yes	Yes	Yes	No (NPD)	No (NM)	No (NM)
Oka et al. 2019; Japan	Yes	Yes	Yes	Yes	Yes	Yes	No (NM)	No (NM)	Yes	Yes	Yes	No (NM)?	Yes	Yes	Yes	Yes	No (NM)	No (NM)	No (NM)
**TAICHI**																			
Elhamrawy et al. 2023; Egypt	No (NM)	No (NM)	No (NM)	No (NM)	No (NM)	No (NM)	No (NM)	No (NM)	Yes	No (NM)	Yes	No (NM)	No (NM)	Yes	No (NM)	No (NM)	No (NM)	No (NM)	No (NM)
Liu and Lei. 2010; China	No (NM)	No (NM)	No (NPD)	No (NPD)	No (NM)	No (NM)	No (NM)	No (NM)	No (NM)	No (NM)	No (NM)	No (NM)	Yes	No (NM)	No (NM)	No (NM)	No (NM)	No (NM)	No (NM)

Yes = Items are included in clinical trials; No = Items are not included in clinical trials, or the items are omitted.

NA= Not applicable; NPD= Not properly detailed; NM= Not mentioned

In some studies, it is specified that the exercise-based intervention has been identical for all study subjects and there has not been any type of personalization. Items 14b and 15 correspond to information that can only be given in the case of individualized exercise. Those where no information can be provided have been scored as NA.

Supplementary File S6. Subgroup meta-analyses and forest plots for physical fatigue.

Study or Subgroup	Experimental Mean	Experimental SD	Experimental Total	Control Mean	Control SD	Control Total	Weight	Std. Mean Difference IV, Fixed, 95% CI
**1.13.1 Qigong**								
Chan et al., 2013	15.9	8	53	20.8	5.7	58	28.1%	-0.71 [-1.09, -0.32]
Chan et al., 2014	16.7	8	65	20.6	5.9	65	33.8%	-0.55 [-0.90, -0.20]
Xie et al., 2022 (a)	4.5	2.8	45	6.7	3.2	44	22.5%	-0.73 [-1.16, -0.30]
Xie et al., 2023	5.9	2.4	19	8.9	2.2	20	8.6%	-1.28 [-1.97, -0.58]
**Subtotal (95% CI)**			**182**			**187**	**92.9%**	**-0.71 [-0.92, -0.50]**
Heterogeneity: Chi² = 3.35, df = 3 (P = 0.34); I² = 11%								
Test for overall effect: Z = 6.56 (P < 0.00001)								
**1.13.2 Yoga**								
Oka et al., 2014	12.3	3.8	15	16.1	3.6	15	7.1%	-1.00 [-1.76, -0.23]
**Subtotal (95% CI)**			**15**			**15**	**7.1%**	**-1.00 [-1.76, -0.23]**
Heterogeneity: Not applicable								
Test for overall effect: Z = 2.56 (P = 0.01)								
**Total (95% CI)**			**197**			**202**	**100.0%**	**-0.73 [-0.93, -0.52]**
Heterogeneity: Chi² = 3.87, df = 4 (P = 0.42); I² = 0%								
Test for overall effect: Z = 7.01 (P < 0.00001)								
Test for subgroup differences: Chi² = 0.52, df = 1 (P = 0.47), I² = 0%								

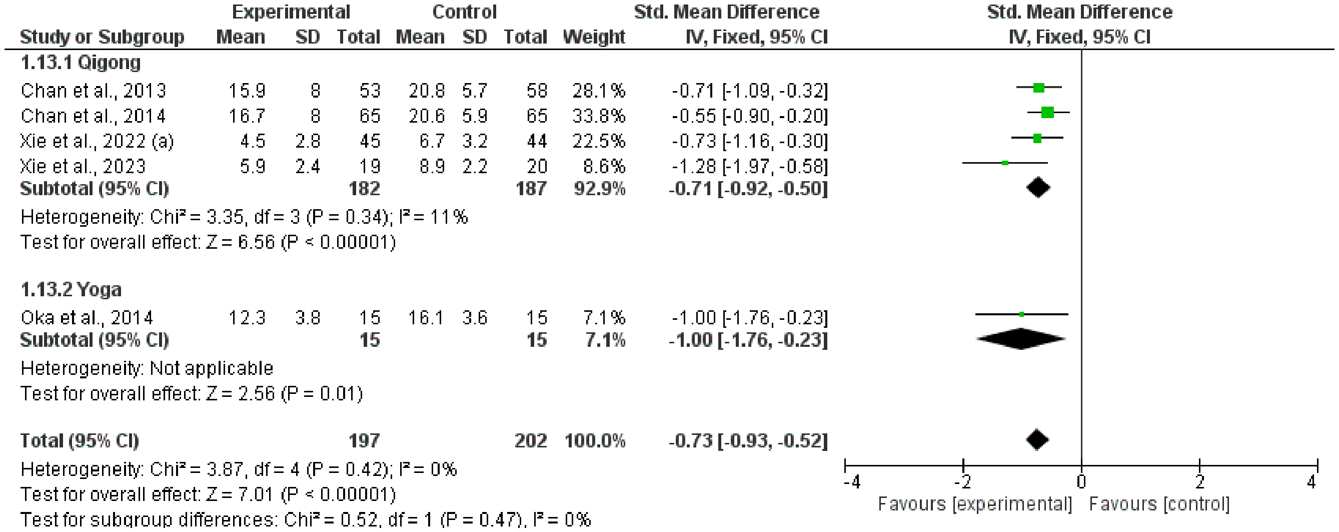

Study or Subgroup	Experimental Mean	Experimental SD	Experimental Total	Control Mean	Control SD	Control Total	Weight	Std. Mean Difference IV, Fixed, 95% CI
**1.14.1 Comparison: no intervention**								
Chan et al., 2013	15.9	8	53	20.8	5.7	58	28.1%	-0.71 [-1.09, -0.32]
Chan et al., 2014	16.7	8	65	20.6	5.9	65	33.8%	-0.55 [-0.90, -0.20]
Oka et al., 2014	12.3	3.8	15	16.1	3.6	15	7.1%	-1.00 [-1.76, -0.23]
**Subtotal (95% CI)**			**133**			**138**	**68.9%**	**-0.66 [-0.91, -0.42]**
Heterogeneity: Chi² = 1.18, df = 2 (P = 0.56); I² = 0%								
Test for overall effect: Z = 5.28 (P < 0.00001)								
**1.14.2 Comparison: other interventions**								
Xie et al., 2022 (a)	4.5	2.8	45	6.7	3.2	44	22.5%	-0.73 [-1.16, -0.30]
Xie et al., 2023	5.9	2.4	19	8.9	2.2	20	8.6%	-1.28 [-1.97, -0.58]
**Subtotal (95% CI)**			**64**			**64**	**31.1%**	**-0.88 [-1.24, -0.51]**
Heterogeneity: Chi² = 1.75, df = 1 (P = 0.19); I² = 43%								
Test for overall effect: Z = 4.71 (P < 0.00001)								
**Total (95% CI)**			**197**			**202**	**100.0%**	**-0.73 [-0.93, -0.52]**
Heterogeneity: Chi² = 3.87, df = 4 (P = 0.42); I² = 0%								
Test for overall effect: Z = 7.01 (P < 0.00001)								
Test for subgroup differences: Chi² = 0.94, df = 1 (P = 0.33), I² = 0%								

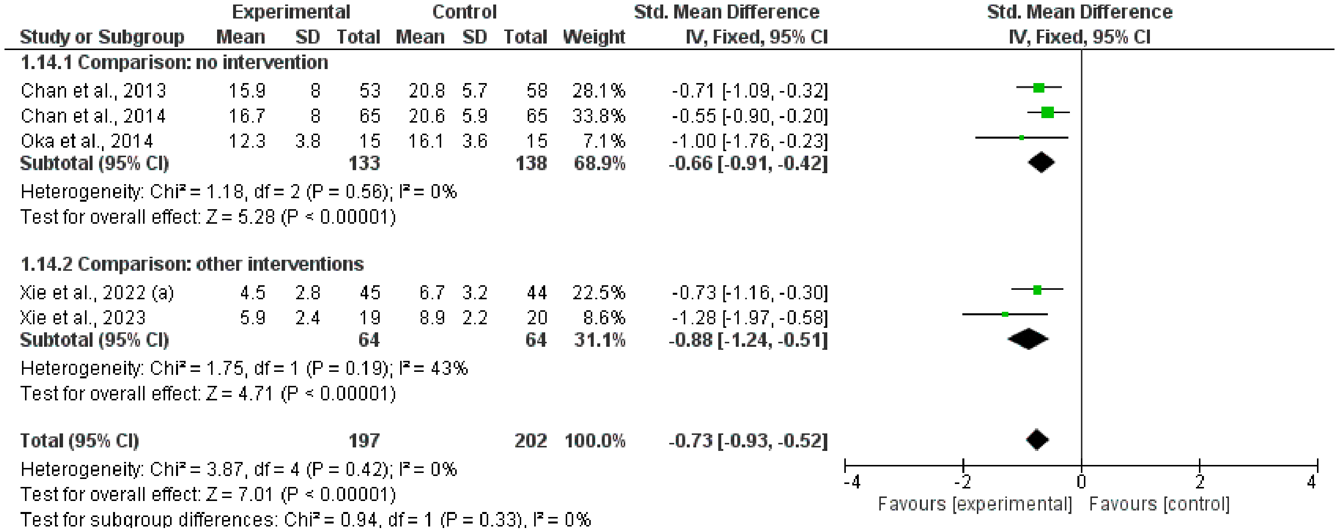

Supplementary File S7. Subgroup meta-analyses and forest plots for mental fatigue.

Study or Subgroup	Experimental Mean	Experimental SD	Experimental Total	Control Mean	Control SD	Control Total	Weight	Std. Mean Difference IV, Fixed, 95% CI
**1.16.1 Qigong**								
Chan et al., 2013	10.6	6.1	53	12.4	4.9	58	28.1%	-0.32 [-0.70, 0.05]
Chan et al., 2014	9	5.4	65	11.7	4.6	65	32.2%	-0.54 [-0.89, -0.19]
Xie et al., 2022 (a)	5.6	2.7	45	6.3	2.7	44	22.7%	-0.26 [-0.67, 0.16]
Xie et al., 2023	6.1	3.8	19	8	5.3	20	9.8%	-0.40 [-1.04, 0.23]
**Subtotal (95% CI)**			**182**			**187**	**92.8%**	**-0.39 [-0.60, -0.18]**
Heterogeneity: Chi² = 1.17, df = 3 (P = 0.76); I² = 0%								
Test for overall effect: Z = 3.70 (P = 0.0002)								
**1.16.2 Yoga**								
Oka et al., 2014	6.9	4.4	15	9.7	3.1	15	7.2%	-0.72 [-1.46, 0.03]
**Subtotal (95% CI)**			**15**			**15**	**7.2%**	**-0.72 [-1.46, 0.03]**
Heterogeneity: Not applicable								
Test for overall effect: Z = 1.89 (P = 0.06)								
**Total (95% CI)**			**197**			**202**	**100.0%**	**-0.41 [-0.61, -0.21]**
Heterogeneity: Chi² = 1.86, df = 4 (P = 0.76); I² = 0%								
Test for overall effect: Z = 4.07 (P < 0.0001)								
Test for subgroup differences: Chi² = 0.69, df = 1 (P = 0.41), I² = 0%								

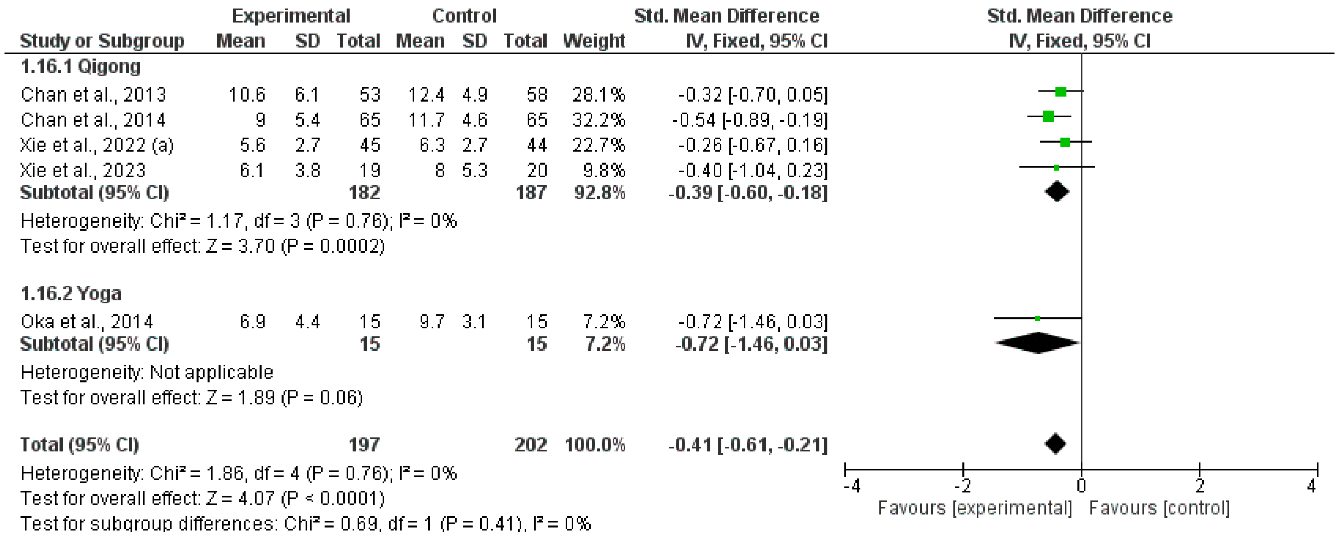

Study or Subgroup	Experimental Mean	Experimental SD	Experimental Total	Control Mean	Control SD	Control Total	Weight	Std. Mean Difference IV, Fixed, 95% CI
**1.17.1 Comparison: no intervention**								
Chan et al., 2013	10.6	6.1	53	12.4	4.9	58	28.1%	-0.32 [-0.70, 0.05]
Chan et al., 2014	9	5.4	65	11.7	4.6	65	32.2%	-0.54 [-0.89, -0.19]
Oka et al., 2014	6.9	4.4	15	9.7	3.1	15	7.2%	-0.72 [-1.46, 0.03]
**Subtotal (95% CI)**			**133**			**138**	**67.5%**	**-0.47 [-0.71, -0.22]**
Heterogeneity: Chi² = 1.13, df = 2 (P = 0.57); I² = 0%								
Test for overall effect: Z = 3.78 (P = 0.0002)								
**1.17.2 Comparison: other interventions**								
Xie et al., 2022 (a)	5.6	2.7	45	6.3	2.7	44	22.7%	-0.26 [-0.67, 0.16]
Xie et al., 2023	6.1	3.8	19	8	5.3	20	9.8%	-0.40 [-1.04, 0.23]
**Subtotal (95% CI)**			**64**			**64**	**32.5%**	**-0.30 [-0.65, 0.05]**
Heterogeneity: Chi² = 0.14, df = 1 (P = 0.71); I² = 0%								
Test for overall effect: Z = 1.69 (P = 0.09)								
**Total (95% CI)**			**197**			**202**	**100.0%**	**-0.41 [-0.61, -0.21]**
Heterogeneity: Chi² = 1.86, df = 4 (P = 0.76); I² = 0%								
Test for overall effect: Z = 4.07 (P < 0.0001)								
Test for subgroup differences: Chi² = 0.59, df = 1 (P = 0.44), I² = 0%								

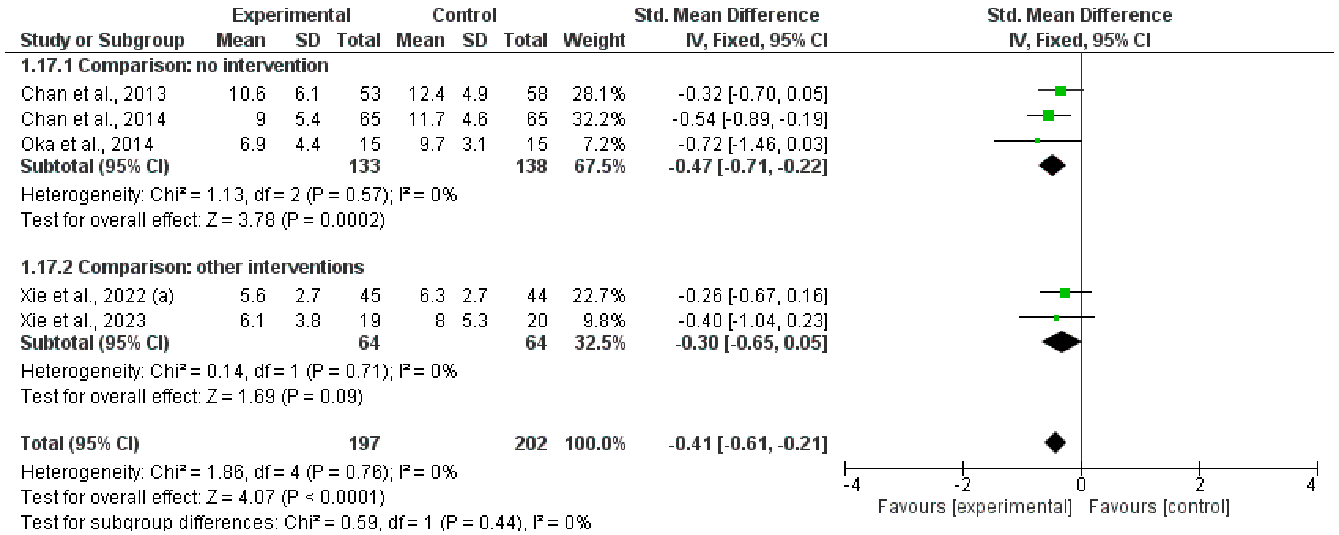

Supplementary File S8. Subgroup meta-analyses and forest plots for overall fatigue.

Study or Subgroup	Experimental Mean	Experimental SD	Experimental Total	Control Mean	Control SD	Control Total	Weight	Std. Mean Difference IV, Fixed, 95% CI
**1.10.1 Qigong**								
Chan et al., 2013	26.6	13.6	53	33.2	9.6	58	25.4%	-0.56 [-0.94, -0.18]
Chan et al., 2014	25.6	12.6	65	32.3	9.7	65	29.7%	-0.59 [-0.94, -0.24]
Xie et al., 2022 (a)	6.5	2.9	45	7.3	3.4	44	21.1%	-0.25 [-0.67, 0.17]
Xie et al., 2023	7.4	2.6	19	8.8	3.3	20	9.0%	-0.46 [-1.10, 0.18]
**Subtotal (95% CI)**			**182**			**187**	**85.2%**	**-0.48 [-0.69, -0.28]**
Heterogeneity: Chi² = 1.72, df = 3 (P = 0.63); I² = 0%								
Test for overall effect: Z = 4.58 (P < 0.00001)								
**1.10.2 Yoga**								
Oka et al., 2014	19.2	7.5	15	25.8	5.9	15	6.3%	-0.95 [-1.71, -0.19]
**Subtotal (95% CI)**			**15**			**15**	**6.3%**	**-0.95 [-1.71, -0.19]**
Heterogeneity: Not applicable								
Test for overall effect: Z = 2.45 (P = 0.01)								
**1.10.3 Tai Chi**								
Elhamrawy et al., 2023	44.9	3.8	18	43.7	2.1	18	8.4%	0.38 [-0.28, 1.04]
**Subtotal (95% CI)**			**18**			**18**	**8.4%**	**0.38 [-0.28, 1.04]**
Heterogeneity: Not applicable								
Test for overall effect: Z = 1.13 (P = 0.26)								
**Total (95% CI)**			**215**			**220**	**100.0%**	**-0.44 [-0.63, -0.25]**
Heterogeneity: Chi² = 9.60, df = 5 (P = 0.09); I² = 48%								
Test for overall effect: Z = 4.51 (P < 0.00001)								
Test for subgroup differences: Chi² = 7.88, df = 2 (P = 0.02), I² = 74.6%								

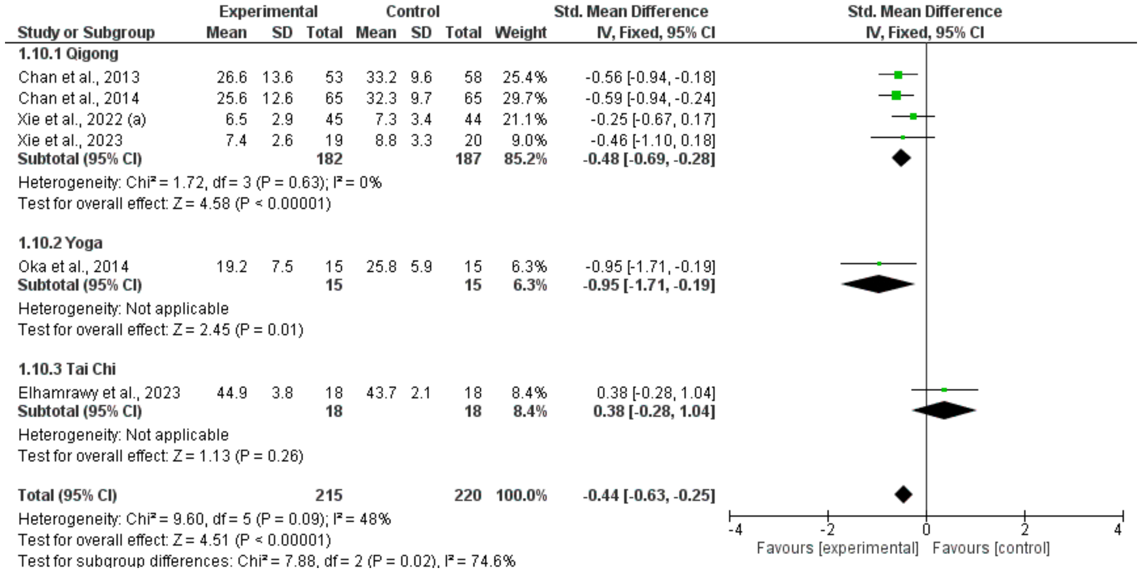

Study or Subgroup	Experimental Mean	Experimental SD	Experimental Total	Control Mean	Control SD	Control Total	Weight	Std. Mean Difference IV, Fixed, 95% CI
**1.11.1 Comparison: no intervention**								
Chan et al., 2013	26.6	13.6	53	33.2	9.6	58	27.8%	-0.56 [-0.94, -0.18]
Chan et al., 2014	25.6	12.6	65	32.3	9.7	65	32.4%	-0.59 [-0.94, -0.24]
Oka et al., 2014	19.2	7.5	15	25.8	5.9	15	6.9%	-0.95 [-1.71, -0.19]
**Subtotal (95% CI)**			**133**			**138**	**67.1%**	**-0.62 [-0.86, -0.37]**
Heterogeneity: Chi² = 0.85, df = 2 (P = 0.66); I² = 0%								
Test for overall effect: Z = 4.95 (P < 0.00001)								
**1.11.2 Comparison: other interventions**								
Xie et al., 2022 (a)	6.5	2.9	45	7.3	3.4	44	23.0%	-0.25 [-0.67, 0.17]
Xie et al., 2023	7.4	2.6	19	8.8	3.3	20	9.9%	-0.46 [-1.10, 0.18]
**Subtotal (95% CI)**			**64**			**64**	**32.9%**	**-0.31 [-0.66, 0.04]**
Heterogeneity: Chi² = 0.29, df = 1 (P = 0.59); I² = 0%								
Test for overall effect: Z = 1.76 (P = 0.08)								
**Total (95% CI)**			**197**			**202**	**100.0%**	**-0.52 [-0.72, -0.32]**
Heterogeneity: Chi² = 3.07, df = 4 (P = 0.55); I² = 0%								
Test for overall effect: Z = 5.06 (P < 0.00001)								
Test for subgroup differences: Chi² = 1.94, df = 1 (P = 0.16), I² = 48.4%								

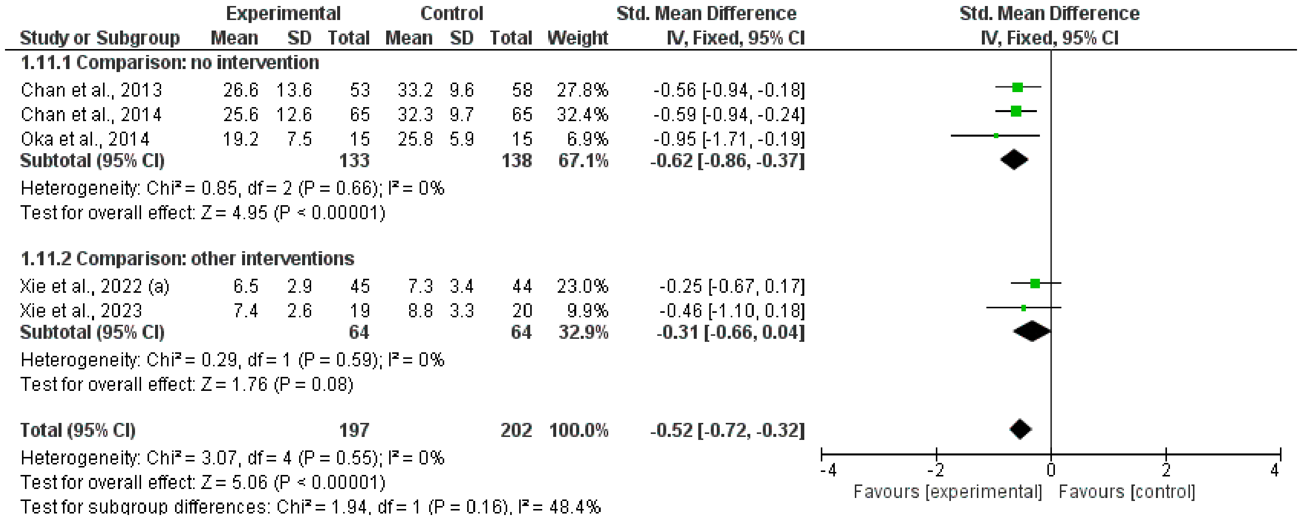

Supplementary File S9. Funnel diagrams for physical, mental and overall fatigue.

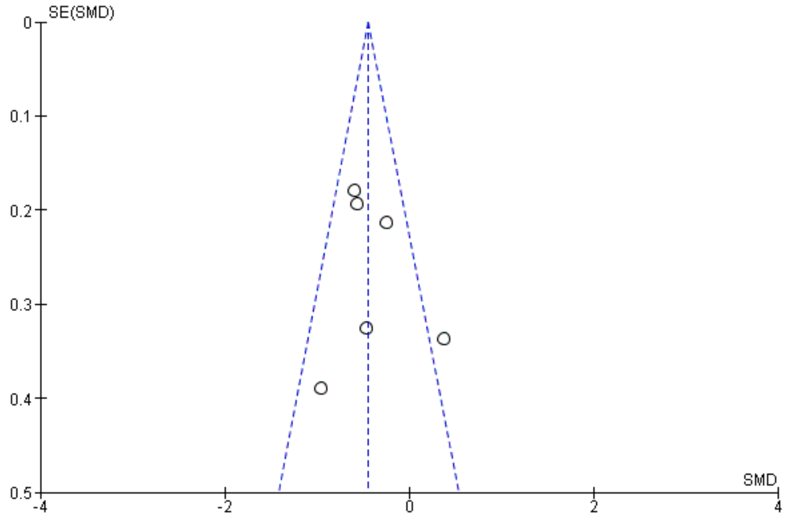

Funnel diagram for total fatigue

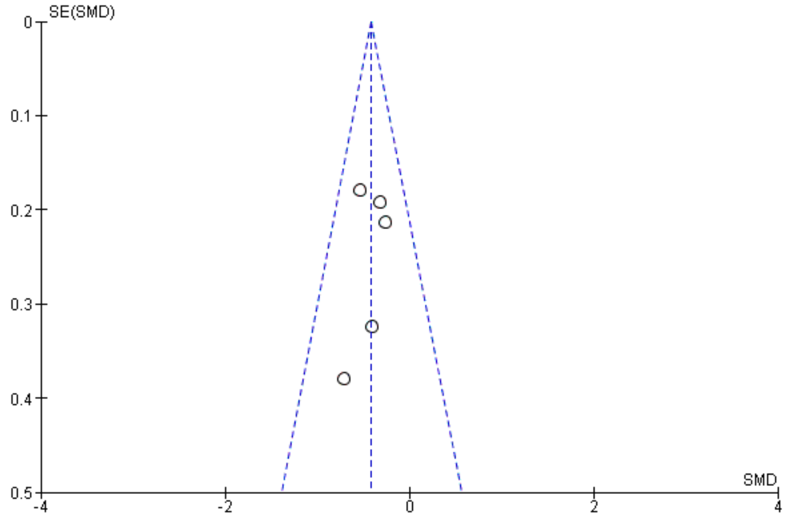

Funnel diagram for mental fatigue

Funnel diagram for physical fatigue

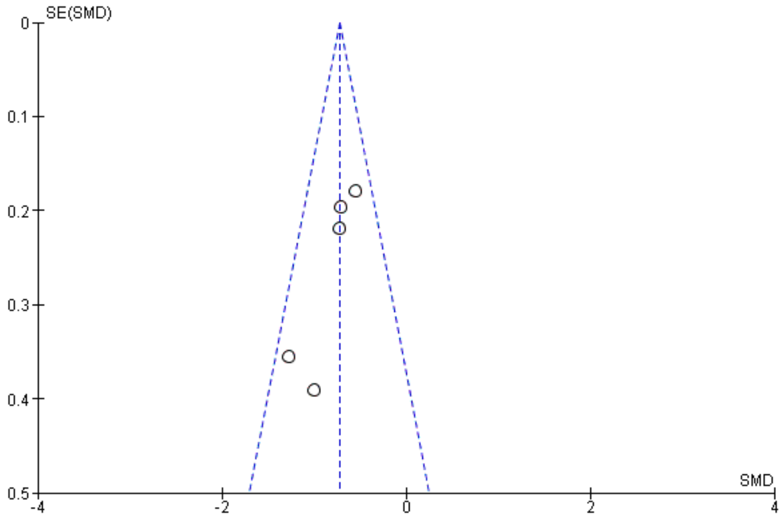

Supplementary File S10. Meta-analysis and forest plot for anxiety symptoms.

	Experimental			Control				Mean Difference
Study or Subgroup	Mean	SD	Total	Mean	SD	Total	Weight	IV, Fixed, 95% CI
Chan et al., 2013	8.7	3.2	53	9	4	58	27.0%	-0.30 [-1.64, 1.04]
Chan et al., 2014	8.5	4	65	10.4	4	65	25.7%	-1.90 [-3.28, -0.52]
Oka et al., 2019	6.9	4	15	9	3.2	15	7.2%	-2.10 [-4.69, 0.49]
Xie et al., 2022 (a)	4.1	2.1	45	5.7	3.1	44	40.0%	-1.60 [-2.70, -0.50]
**Total (95% CI)**			**178**			**182**	**100.0%**	**-1.36 [-2.06, -0.66]**

Heterogeneity: Chi² = 3.48, df = 3 (P = 0.32); I² = 14%
Test for overall effect: Z = 3.83 (P = 0.0001)

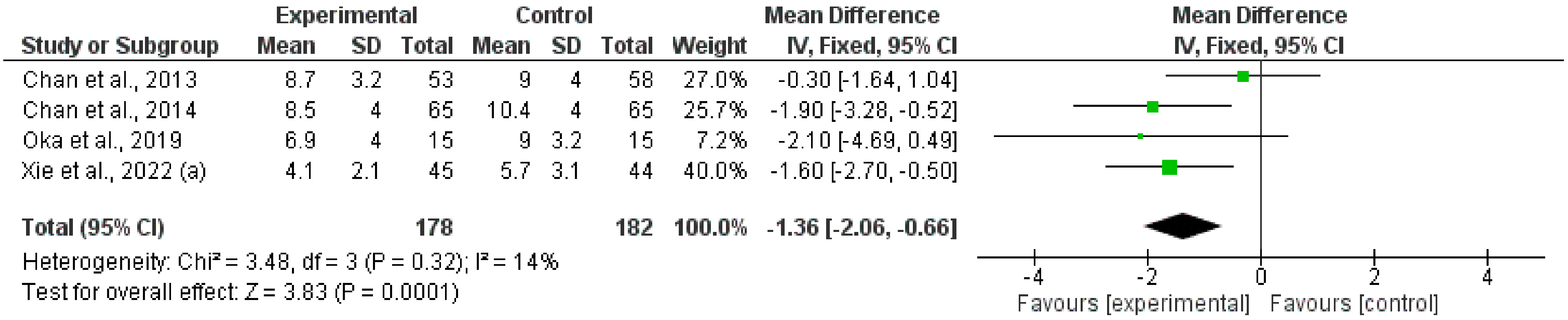

Supplementary File S11. Subgroup meta-analyses and forest plots for anxiety symptoms.

	Experimental			Control				Mean Difference
Study or Subgroup	Mean	SD	Total	Mean	SD	Total	Weight	IV, Fixed, 95% CI
**1.2.1 Qigong**								
Chan et al., 2013	8.7	3.2	53	9	4	58	27.0%	-0.30 [-1.64, 1.04]
Chan et al., 2014	8.5	4	65	10.4	4	65	25.7%	-1.90 [-3.28, -0.52]
Xie et al., 2022 (a)	4.1	2.1	45	5.7	3.1	44	40.0%	-1.60 [-2.70, -0.50]
**Subtotal (95% CI)**			**163**			**167**	**92.8%**	**-1.30 [-2.03, -0.58]**
Heterogeneity: Chi² = 3.15, df = 2 (P = 0.21); I² = 36%								
Test for overall effect: Z = 3.53 (P = 0.0004)								
**1.2.2 Yoga**								
Oka et al., 2019	6.9	4	15	9	3.2	15	7.2%	-2.10 [-4.69, 0.49]
**Subtotal (95% CI)**			**15**			**15**	**7.2%**	**-2.10 [-4.69, 0.49]**
Heterogeneity: Not applicable								
Test for overall effect: Z = 1.59 (P = 0.11)								
**Total (95% CI)**			**178**			**182**	**100.0%**	**-1.36 [-2.06, -0.66]**
Heterogeneity: Chi² = 3.48, df = 3 (P = 0.32); I² = 14%								
Test for overall effect: Z = 3.83 (P = 0.0001)								
Test for subgroup differences: Chi² = 0.34, df = 1 (P = 0.56), I² = 0%								

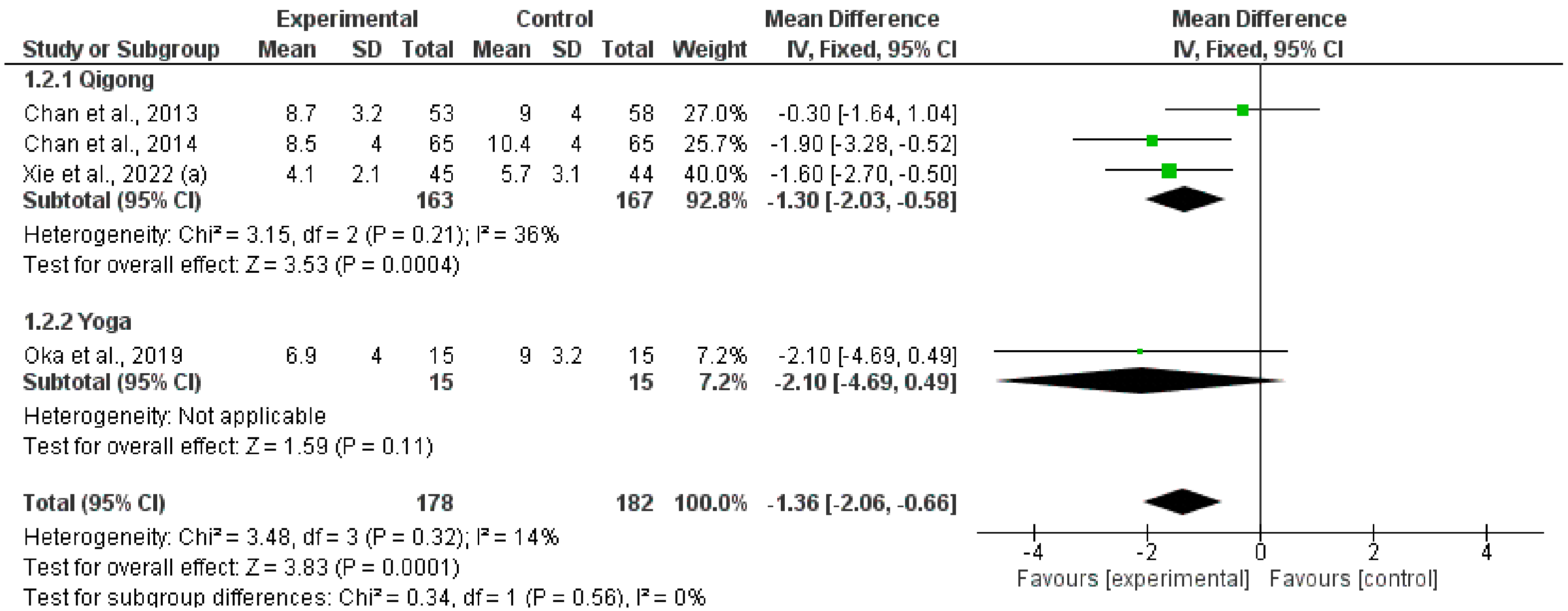

Study or Subgroup	Experimental Mean	Experimental SD	Experimental Total	Control Mean	Control SD	Control Total	Weight	Mean Difference IV, Fixed, 95% CI
**1.3.1 Comparison: no intervention**								
Chan et al., 2013	8.7	3.2	53	9	4	58	27.0%	-0.30 [-1.64, 1.04]
Chan et al., 2014	8.5	4	65	10.4	4	65	25.7%	-1.90 [-3.28, -0.52]
Oka et al., 2019	6.9	4	15	9	3.2	15	7.2%	-2.10 [-4.69, 0.49]
**Subtotal (95% CI)**			**133**			**138**	**60.0%**	**-1.20 [-2.10, -0.30]**
Heterogeneity: Chi² = 3.19, df = 2 (P = 0.20); I² = 37%								
Test for overall effect: Z = 2.62 (P = 0.009)								
**1.3.2 Comparison: other interventions**								
Xie et al., 2022 (a)	4.1	2.1	45	5.7	3.1	44	40.0%	-1.60 [-2.70, -0.50]
**Subtotal (95% CI)**			**45**			**44**	**40.0%**	**-1.60 [-2.70, -0.50]**
Heterogeneity: Not applicable								
Test for overall effect: Z = 2.84 (P = 0.004)								
**Total (95% CI)**			**178**			**182**	**100.0%**	**-1.36 [-2.06, -0.66]**
Heterogeneity: Chi² = 3.48, df = 3 (P = 0.32); I² = 14%								
Test for overall effect: Z = 3.83 (P = 0.0001)								
Test for subgroup differences: Chi² = 0.30, df = 1 (P = 0.59), I² = 0%								

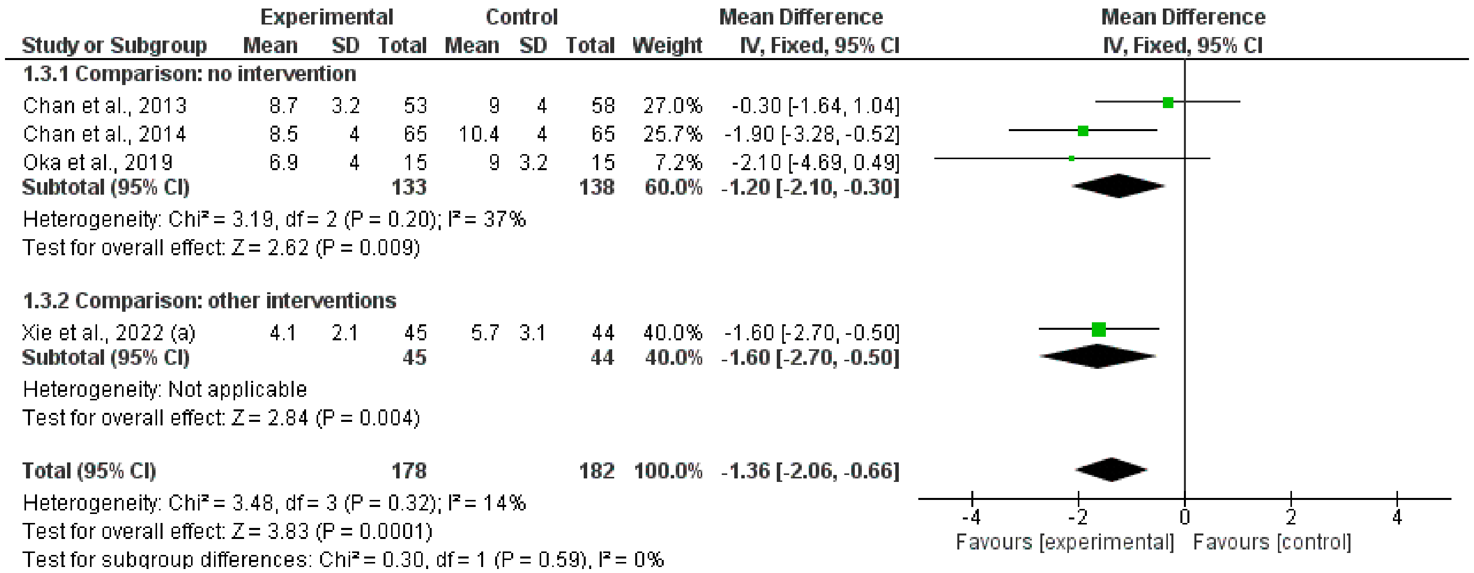

Supplementary File S12. Funnel diagram for anxiety symptoms.

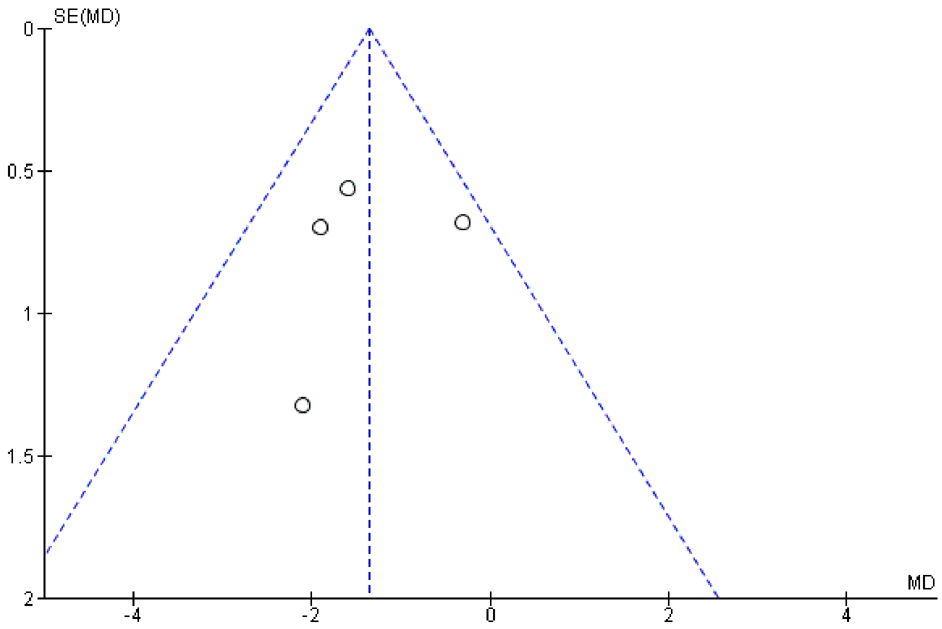

Supplementary file S13. Meta-analysis and forest plot for depressive symptoms.

	Experimental			Control				Mean Difference
**Study or Subgroup**	**Mean**	**SD**	**Total**	**Mean**	**SD**	**Total**	**Weight**	**IV, Fixed, 95% CI**
Chan et al., 2013	7.7	3.2	53	9.8	4.1	58	28.1%	-2.10 [-3.46, -0.74]
Chan et al., 2014	6.6	3.7	65	8.8	3.9	65	30.5%	-2.20 [-3.51, -0.89]
Oka et al., 2019	5.7	5.4	15	10.1	3.8	15	4.7%	-4.40 [-7.74, -1.06]
Xie et al., 2022 (a)	3.4	2.6	45	4.7	3.1	44	36.8%	-1.30 [-2.49, -0.11]
**Total (95% CI)**			**178**			**182**	**100.0%**	**-1.94 [-2.67, -1.22]**

Heterogeneity: Chi² = 3.40, df = 3 (P = 0.33); I² = 12%
Test for overall effect: Z = 5.28 (P < 0.00001)

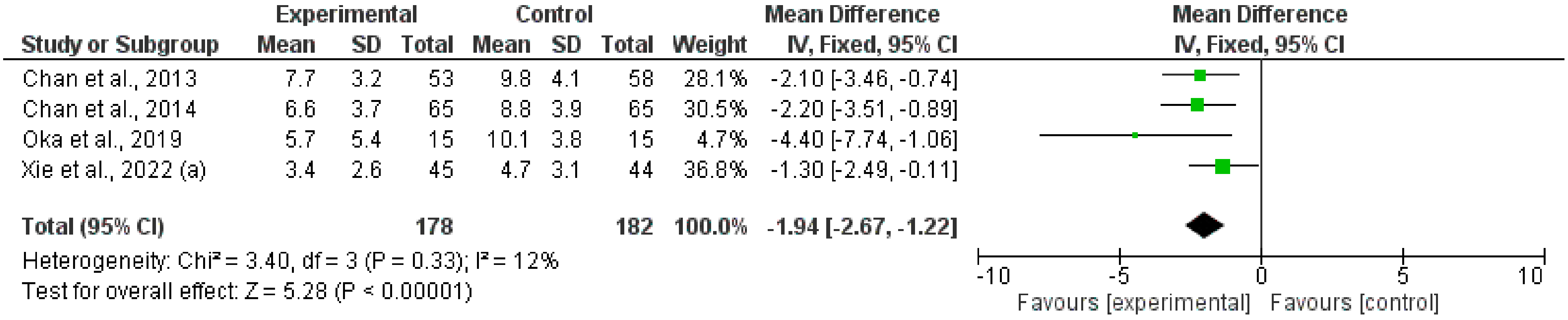

Supplementary File S14. Subgroup meta-analyses and forest plots for depressive symptoms.

Study or Subgroup	Experimental Mean	Experimental SD	Experimental Total	Control Mean	Control SD	Control Total	Weight	Mean Difference IV, Fixed, 95% CI
**1.5.1 Qigong**								
Chan et al., 2013	7.7	3.2	53	9.8	4.1	58	28.1%	-2.10 [-3.46, -0.74]
Chan et al., 2014	6.6	3.7	65	8.8	3.9	65	30.5%	-2.20 [-3.51, -0.89]
Xie et al., 2022 (a)	3.4	2.6	45	4.7	3.1	44	36.8%	-1.30 [-2.49, -0.11]
**Subtotal (95% CI)**			**163**			**167**	**95.3%**	**-1.82 [-2.56, -1.08]**
Heterogeneity: Chi² = 1.22, df = 2 (P = 0.54); I² = 0%								
Test for overall effect: Z = 4.84 (P < 0.00001)								
**1.5.2 Yoga**								
Oka et al., 2019	5.7	5.4	15	10.1	3.8	15	4.7%	-4.40 [-7.74, -1.06]
**Subtotal (95% CI)**			**15**			**15**	**4.7%**	**-4.40 [-7.74, -1.06]**
Heterogeneity: Not applicable								
Test for overall effect: Z = 2.58 (P = 0.010)								
**Total (95% CI)**			**178**			**182**	**100.0%**	**-1.94 [-2.67, -1.22]**
Heterogeneity: Chi² = 3.40, df = 3 (P = 0.33); I² = 12%								
Test for overall effect: Z = 5.28 (P < 0.00001)								
Test for subgroup differences: Chi² = 2.18, df = 1 (P = 0.14), I² = 54.1%								

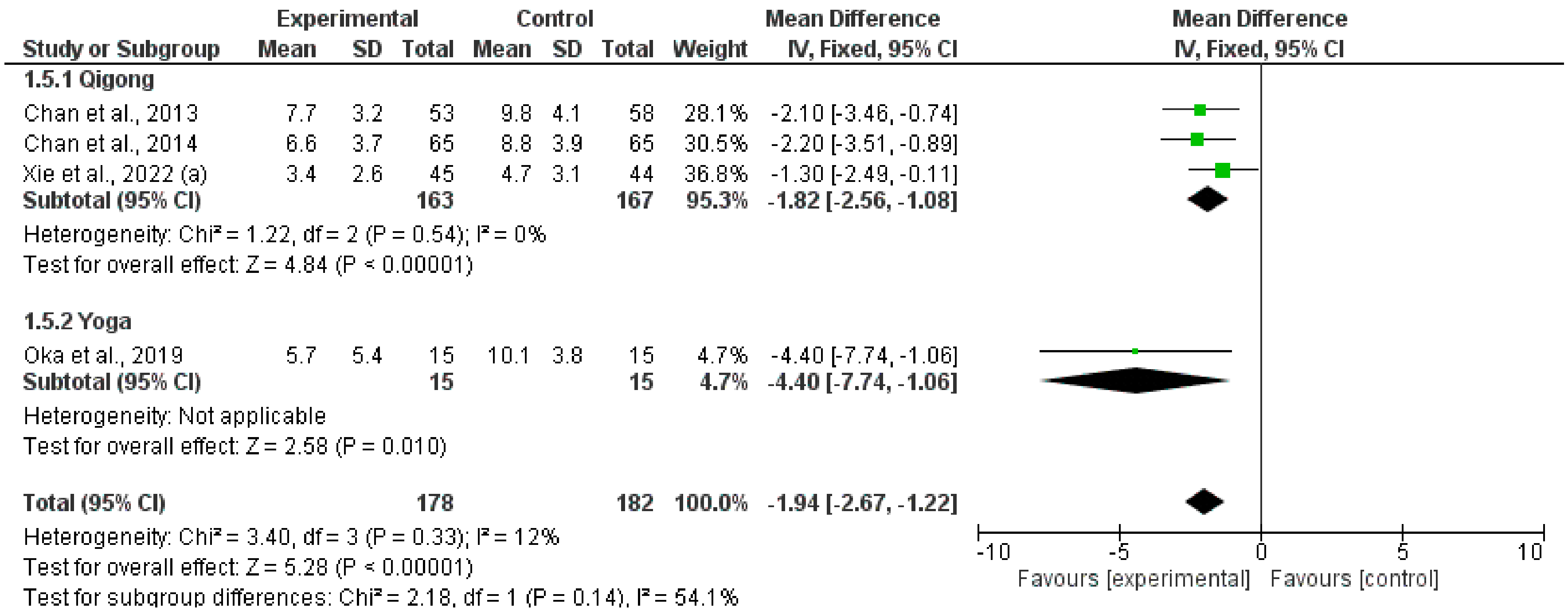

Study or Subgroup	Experimental Mean	Experimental SD	Experimental Total	Control Mean	Control SD	Control Total	Weight	Mean Difference IV, Fixed, 95% CI
**1.6.1 Comparison: no intervention**								
Chan et al., 2013	7.7	3.2	53	9.8	4.1	58	28.1%	-2.10 [-3.46, -0.74]
Chan et al., 2014	6.6	3.7	65	8.8	3.9	65	30.5%	-2.20 [-3.51, -0.89]
Oka et al., 2019	5.7	5.4	15	10.1	3.8	15	4.7%	-4.40 [-7.74, -1.06]
**Subtotal (95% CI)**			**133**			**138**	**63.2%**	**-2.32 [-3.23, -1.41]**
Heterogeneity: Chi² = 1.62, df = 2 (P = 0.44); I² = 0%								
Test for overall effect: Z = 5.01 (P < 0.00001)								
**1.6.2 Comparison: other interventions**								
Xie et al., 2022 (a)	3.4	2.6	45	4.7	3.1	44	36.8%	-1.30 [-2.49, -0.11]
**Subtotal (95% CI)**			**45**			**44**	**36.8%**	**-1.30 [-2.49, -0.11]**
Heterogeneity: Not applicable								
Test for overall effect: Z = 2.14 (P = 0.03)								
**Total (95% CI)**			**178**			**182**	**100.0%**	**-1.94 [-2.67, -1.22]**
Heterogeneity: Chi² = 3.40, df = 3 (P = 0.33); I² = 12%								
Test for overall effect: Z = 5.28 (P < 0.00001)								
Test for subgroup differences: Chi² = 1.78, df = 1 (P = 0.18), I² = 43.7%								

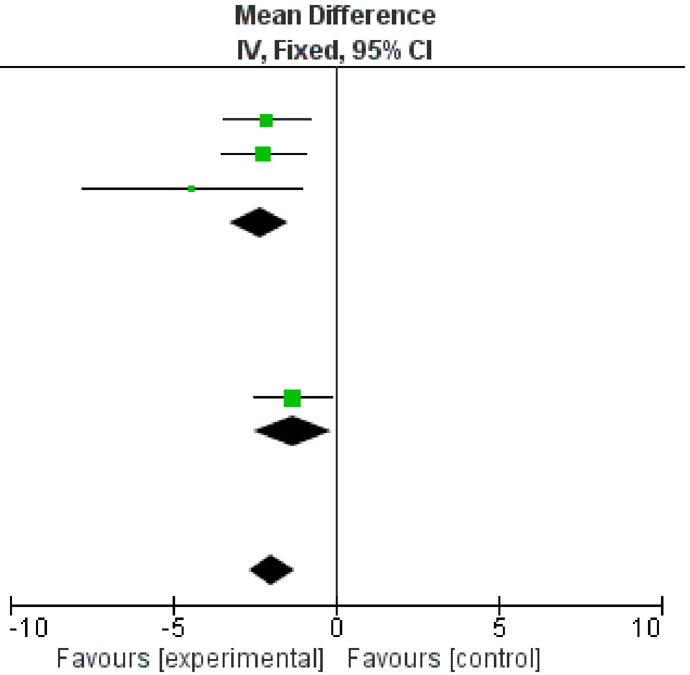

Supplementary File S15. Funnel diagram for depressive symptoms.

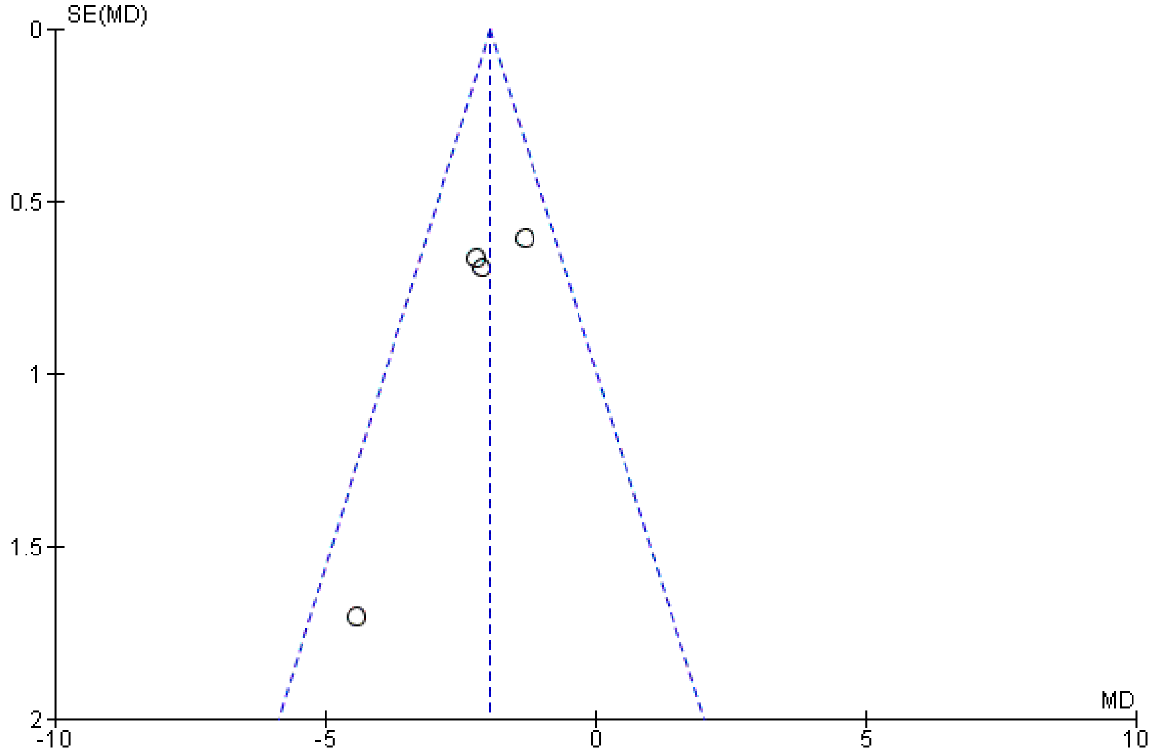

Supplementary File S16. Meta-analysis and forest plot for quality of sleep.

Study or Subgroup	Experimental Mean	Experimental SD	Experimental Total	Control Mean	Control SD	Control Total	Weight	Mean Difference IV, Fixed, 95% CI
Chan et al., 2013	8.2	3.4	65	9.5	3.7	65	32.3%	-1.30 [-2.52, -0.08]
Xie et al., 2022 (a)	4.2	2.1	45	5.3	2.4	44	54.8%	-1.10 [-2.04, -0.16]
Xie et al., 2023	4.2	2.6	19	5.6	3.5	20	12.9%	-1.40 [-3.33, 0.53]
**Total (95% CI)**			**129**			**129**	**100.0%**	**-1.20 [-1.90, -0.51]**

Heterogeneity: Chi² = 0.11, df = 2 (P = 0.95); I² = 0%

Test for overall effect: Z = 3.40 (P = 0.0007)

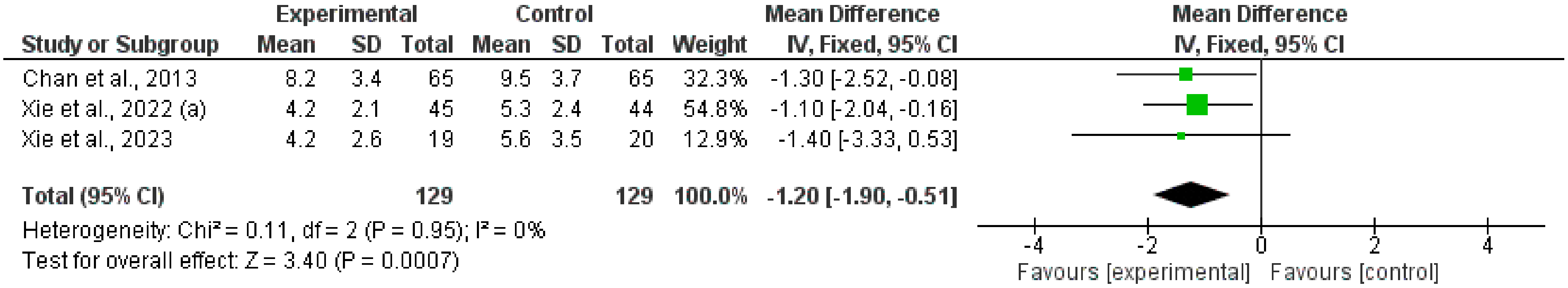

Supplementary File S17. Subgroup meta-analysis and forest plot for quality of sleep.

Study or Subgroup	Experimental Mean	Experimental SD	Experimental Total	Control Mean	Control SD	Control Total	Weight	Mean Difference IV, Fixed, 95% CI
**1.8.1 Comparison: no intervention**								
Chan et al., 2014	8.2	3.4	65	9.5	3.7	65	32.3%	-1.30 [-2.52, -0.08]
**Subtotal (95% CI)**			**65**			**65**	**32.3%**	**-1.30 [-2.52, -0.08]**
Heterogeneity: Not applicable								
Test for overall effect: Z = 2.09 (P = 0.04)								
**1.8.2 Comparison: other interventions**								
Xie et al., 2022 (a)	4.2	2.1	45	5.3	2.4	44	54.8%	-1.10 [-2.04, -0.16]
Xie et al., 2023	4.2	2.6	19	5.6	3.5	20	12.9%	-1.40 [-3.33, 0.53]
**Subtotal (95% CI)**			**64**			**64**	**67.7%**	**-1.16 [-2.00, -0.31]**
Heterogeneity: Chi² = 0.08, df = 1 (P = 0.78); I² = 0%								
Test for overall effect: Z = 2.69 (P = 0.007)								
**Total (95% CI)**			**129**			**129**	**100.0%**	**-1.20 [-1.90, -0.51]**
Heterogeneity: Chi² = 0.11, df = 2 (P = 0.95); I² = 0%								
Test for overall effect: Z = 3.40 (P = 0.0007)								
Test for subgroup differences: Chi² = 0.04, df = 1 (P = 0.85), I² = 0%								

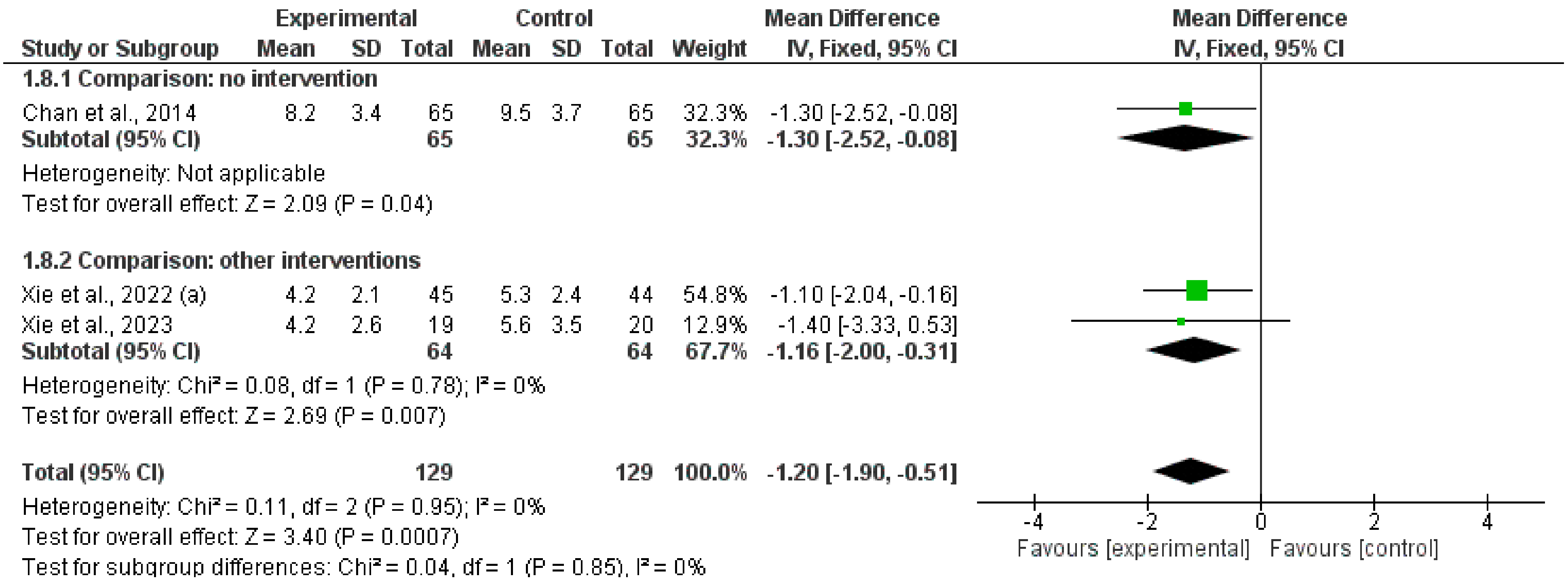

Supplementary File S18. Funnel diagram for quality of sleep.

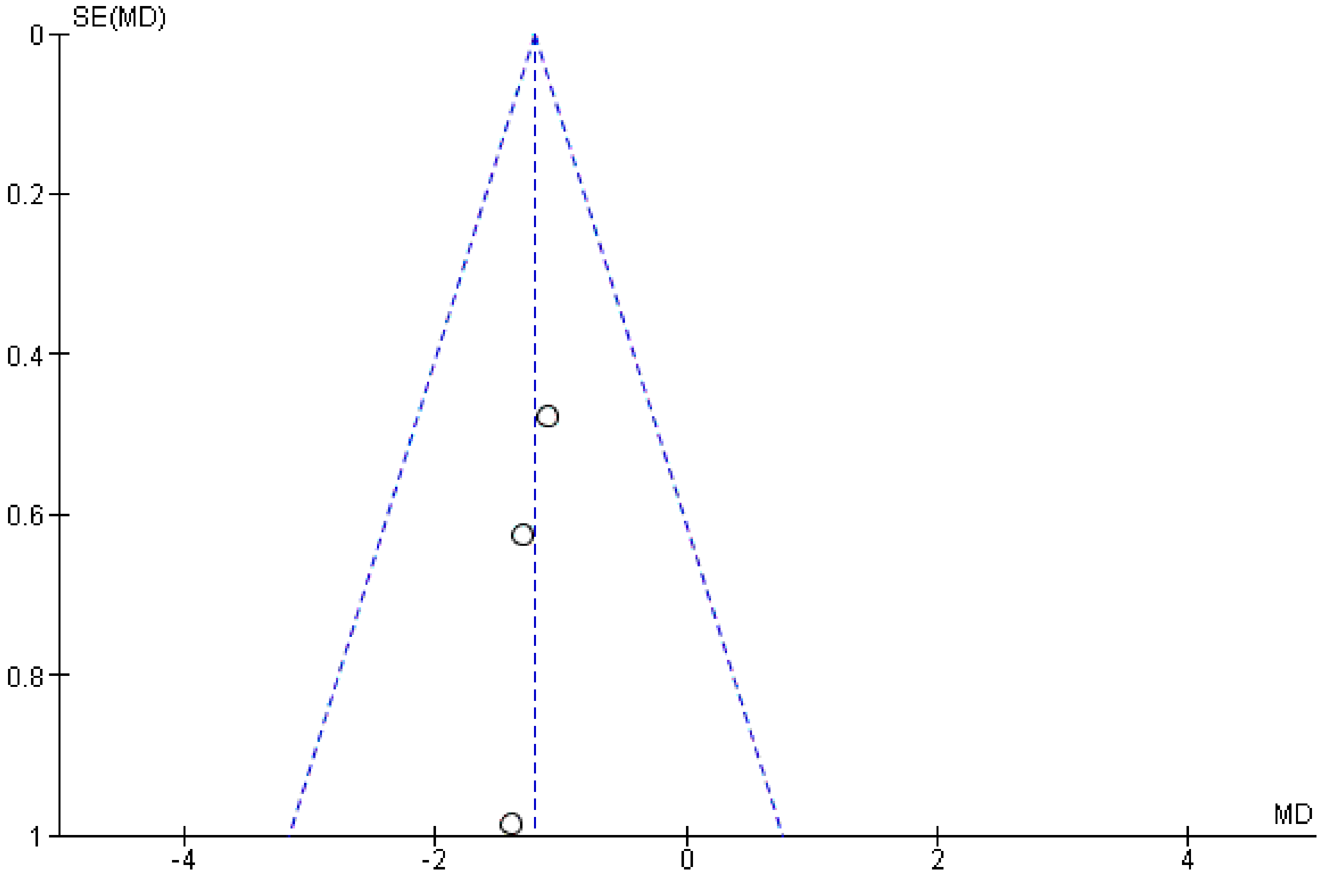

Supplement: Supplementary file 1 [file healthcare-12-02020-s001.zip › healthcare-3213642-supplementary.pdf]
